# NDR2 kinase contributes to cell invasion and cytokinesis defects induced by the inactivation of RASSF1A tumor-suppressor gene in lung cancer cells

**DOI:** 10.1186/s13046-019-1145-8

**Published:** 2019-04-12

**Authors:** Maureen Keller, Fatéméh Dubois, Sylvain Teulier, Alexandre P. J. Martin, Jérôme Levallet, Elodie Maille, Solenn Brosseau, Nicolas Elie, Alexander Hergovich, Emmanuel Bergot, Jacques Camonis, Gérard Zalcman, Guénaëlle Levallet

**Affiliations:** 1 Normandie University, UNICAEN, UMR 1086 INSERM, F-14032 Caen, France; 2 Normandie University, UNICAEN, UPRES-EA-2608, F-14032 Caen, France; 3 Normandie University, UNICAEN, CEA, CNRS, ISTCT/CERVOxy group, GIP CYCERON, Avenue H.Becquerel- 14074, F-14000 Caen, France; 4 Service d’Anatomie et Cytologie Pathologique, CHU de Caen, F-14033 Caen, France; 5 U830 INSERM, “Génétique et Biologie des cancers” Centre de Recherche, Institut Curie, Paris, France; 6 Service d’oncologie thoracique, CIC 1425, Hôpital Bichat-Claude Bernard, AP-HP, Université Paris-Diderot, Paris, France; 7 Normandie Univ, UNICAEN, SFR ICORE, Plateau CMABio3, F-14032 Caen, France; 8 University College London Cancer Institute, WC1E 6BT, London, UK; 9 Service de Pneumologie-Oncologie thoracique, CHU de Caen, F-14033 Caen, France

**Keywords:** RASSF1A, GEF-H1, YAP, NDR2 kinase, Lung cancer

## Abstract

**Background:**

RASSF1A, a tumor suppressor gene, is frequently inactivated in lung cancer leading to a YAP-dependent epithelial-mesenchymal transition (EMT). Such effects are partly due to the inactivation of the anti-migratory RhoB GTPase via the inhibitory phosphorylation of GEF-H1, the GDP/GTP exchange factor for RhoB. However, the kinase responsible for RhoB/GEF-H1 inactivation in RASSF1A-depleted cells remained unknown.

**Methods:**

NDR1/2 inactivation by siRNA or shRNA effects on epithelial-mesenchymal transition, invasion, xenograft formation and growth in SCID−/− Beige mice, apoptosis, proliferation, cytokinesis, YAP/TAZ activation were investigated upon RASSF1A loss in human bronchial epithelial cells (HBEC).

**Results:**

We demonstrate here that depletion of the YAP-kinases NDR1/2 reverts migration and metastatic properties upon RASSF1A loss in HBEC. We show that NDR2 interacts directly with GEF-H1 (which contains the NDR phosphorylation consensus motif HXRXXS/T), leading to GEF-H1 phosphorylation. We further report that the RASSF1A/NDR2/GEF-H1/RhoB/YAP axis is involved in proper cytokinesis in human bronchial cells, since chromosome proper segregation are NDR-dependent upon RASSF1A or GEF-H1 loss in HBEC.

**Conclusion:**

To summarize, our data support a model in which, upon RASSF1A silencing, NDR2 gets activated, phosphorylates and inactivates GEF-H1, leading to RhoB inactivation. This cascade induced by RASSF1A loss in bronchial cells is responsible for metastasis properties, YAP activation and cytokinesis defects.

**Electronic supplementary material:**

The online version of this article (10.1186/s13046-019-1145-8) contains supplementary material, which is available to authorized users.

## Background

RASSF1A [Ras association (RalGDS/AF-6) domain family member 1], a tumor and metastatic suppressor gene, is frequently inactivated and an independent predictor of poor prognosis in resected early-stage non–small cell lung cancer (NSCLC) [1, 2]. This worse prognosis value could be sustained by the disturbance of both Rho GTPases [3, 4], and Hippo signaling pathways [3, 5]. RASSF1A loss in human bronchial epithelial cells (HBEC), actually leads to the inactivation of RhoB [3], a Rho GTPase with anti-cell migratory properties [6] and the nuclear translocation of the transcriptional co-activator YAP, with subsequent epithelial-mesenchymal transition (EMT) favoring cell migration and invasion [3]. RhoB inactivation upon RASSF1A loss is the consequence of Rho guanine nucleotide exchange factor GEF-H1 inactivation by phosphorylation [3]. The kinase leading to GEF-H1 inactivation in RASSF1A-depleted HBEC remained unanswered.

The Hippo kinases, NDR1 (STK38) and NDR2 (STK38L) are shown to both phosphorylate/inactivate YAP, one of the terminal targets of Hippo signaling [7, 8] and phosphorylate/inactivate rabin8, a GEF for Rab GTAPses [9]. We thus examined NDR1/2 in the context of RASSF1A loss and report that NDR1/2 knockdown reverted metastatic properties and YAP activation caused by RASSF1A loss in HBEC. We provide evidence for a NDR2/GEF-H1 interaction, leading to GEF-H1 Ser885 phosphorylation/inactivation, followed by RhoB down-regulation. Given the involvement of GEF-H1 and NDR kinases in cell cycle [10, 11], we investigated cell cycle alterations in RASSF1A-depleted HBEC. We found that RASSF1A knockdown induced mitotic abscission defects, which were reverted upon GEF-H1 overexpression or NDR1/2 depletion. We propose a model in which, upon RASSF1A silencing, NDR2 gets activated, phosphorylates GEF-H1, leading to GEF-H1 inactivation, followed by RhoB inactivation. Consequently, RhoB inactivation leads to the cancer-associated phenotypes observed in RASSF1A-depleted HBEC.

## Methods

### Cell culture and transfection

A previous procedure was followed [3]. Cells, whose main molecular alterations are presented Additional file 1: Table S1, were transfected using Lipofectamine RNAiMAX® (Invitrogen™) with siRNA, plasmid DNA or control mimics (Dharmacon™) (Additional file 1: Table S2). Non phosphorylatable GEF-H1 mutants (S265A, S885A) were generated by mutagenesis (Mutagenex, Inc., Suwanee, USA).

### ShNDR1 or NDR2 and SCID mice xenograft

The experiments were performed according to the European Convention for the Protection of Vertebrates Used for Scientific Purposes (Project # 13256).

Groups of ten, strain 250, 6 weeks-old, male Fox Chase SCID−/− Beige mice from Charles River™ were anaesthetized according to the manufacturer’s recommendations for xenografting. ShControl (shRNA Control, Sigma-Aldrich ShNDR1- (NM_007271.2-875s21c1, 5′-CCGGGTATTAGCCATAGACTCTATTCTCGAGAATAGAGTCTATGGCTAATACTTTTTG-3′, Sigma-Aldrich) or shNDR2- (NM_015000.3-1353s21c1, 5′-CCGGGGCTTGCTTGGCGTAGATAACCTCGAGGTTATCTACGCCAAGCAAGCCTTTTTG-3′, Sigma-Aldrich) infected A549 (RASSF1A null) or H1299 (RASSF1A null) cells suspension (1 × 10^7^ cells/0.1 ml) were injected sub-cutaneously in the left flank of each animal. Mice were monitored for tumor growth thrice a week. Tumors were allowed to grow to 1000 mm3 before euthanasia of the mice. The post-mortem examination included macroscopic description of lungs and liver as described [3]. Tumor xenografts, lungs and liver were rapidly removed and fixed in PFA 4% for histological analysis as described [3].

### Reverse transcription-quantitative real-time-PCR (RT-PCR)

After extraction, RT-PCR was done with each primer sets (Additional file 1: Table S2) as described [3]. RT-PCR data were normalized to the human S16. Relative quantification was calculated using the ΔΔCt method.

### λ-Phosphatase assay and immunoblotting

Whole cell protein extracts were prepared as previously [3]. Proteins (2 μg) were incubated with 400 units of λ-phosphatase (Santa Cruz™) and 2 mM MnCl2 at 30 °C. After 30 min, λ-phosphatase was inactivated at 95 °C for 5 min. Proteins were detected by immunoblotting with primary antibody (Additional file 1: Table S3) diluted at 1:1000 in Tween (0.1%)-TBS buffer and HRP-conjugated secondary antibody and revealed by ECL kit (Promega™).

### Immunofluorescence and image analysis

Cells were fixed and permealized as described [3]. Primary antibodies (Additional file 1: Table S3) were diluted at 1:100. Secondary antibodies (AlexaFluo, Invitrogen™) were added for 1 h. Coverslips were mounted with DAPI (Santa Cruz™), and image captured with high-throughput confocal microscopy (FluoView FV1000, Olympus™).

### Immunohistochemistry

Immunohistochemistry was assayed according standard procedures. Slides were incubated overnight at 4 °C with primary antibodies (Additional file 1: Table S3) diluted at 1:200 then revealed using the Novolink (Leica) kit.

### Wound healing assay

Cells grown onto 24-well Collagen IV coated plates (BD Biocoat™) were treated with mitomycin C (1 μg/ml) 12 h before an artificial “wound” created at 0 h. Photographs were taken (X10) at 0 h and 12 h. The distances traveled were expressed as μm/h.

### Migration 3D & invasion assays

Cells (20 × 10^3^) were added in the top invasion chambers of 24-well transwell plates containing cell culture insert (BD BioCoat Matrigel® Invasion Chamber, BD Biosciences™). At 48 h, migrating cells were stained with crystal violet.

### Bromodeoxyuridine (BrdU) incorporation analysis

Cells labeled with BrdU (1:500 dilution, cell proliferation assay, Millipore) for 24 and 48 h were fixed for 30 min then BrdU was detected using anti-BrdU mouse monoclonal antibody followed by peroxidase-conjugated goat anti-mouse IgG antibody. The colored reaction was quantified using a microplate reader at 450 nm.

### DNA fragmentation assays

Cells (1 × 10^5^) were resuspended in lysis buffer (200 μL) supplied by the manufacturer (Cell Death Detection; Roche). The cytoplasmic fraction (20 μL) was used for the enzyme-linked immunosorbent assay (ELISA). Absorbance at 405 nm was determined with a microplate reader.

### Viability

Cell viability was assessed by staining cells with Trypan blue solution (5%) and numbering non-viable (blue) cells under microscope (× 20) in four 1 × 1 mm squares of one chamber and determining the average number of cells per square.

### Co-immunoprecipitation and GTP-rho, NDR2/GEF-H1 pull-down assays

Cells were lysed in chilled immunoprecipitation buffer and the cleared lysate (500 μg) incubated with 3 μg of the indicated antibody and 30 μL of protein-A agarose beads (Repligen) in 1 mL of buffer. Beads were resuspended in 30 μL of 2X Laemmli buffer and subjected to Western blotting.

For GTP-pulldown assays, cell lysates were incubated with beads glutathione-S-transferase (GST)-Rhotekin Rho binding domain (RBD), GST-NDR1 or GST-NDR2 (Carna Biosciences, Japan), then precipitates analyzed by Western blotting using anti-RhoB, anti-GEF-H1 or anti-S885phospho-GEF-H1 antibodies.

### Live cell imaging and analysis

Cells were grown on 35-mm coverglass bottom dishes (MatTek). The microscope was equipped with an open chamber (Pecon) equilibrated in 5% CO_2_ and maintained at 37 °C. Images were taken at 2-min intervals with a × 20 or × 60 objective using a RTKE camera controlled by the Micromanager software. Video analysis was performed by ≥ImageJ software.

### Statistical analysis

Data are means ± SEM (*n* ≥ 3). Statistical differences were determined by one-way analysis of variance (ANOVA) followed by Dunnett’s Multiple Comparison Test (GraphPad Software, Inc. USA). Statistical significance was set at *p* ≤ 0.05. Chi2 test was used to test correlation between events. We used kpm.plot.com online software to compute the mRNA prognostic analyses in 681 Stage I-to-III patients, with gene-expression data and OS information downloaded from the GEO (Affymetrix microarrays only), EGA, and TCGA databases.

## Results

### Depletion of the NDR1/2 kinases reverts the migratory and metastatic phenotypes induced by RASSF1A loss in HBEC

**Fig. 1:**
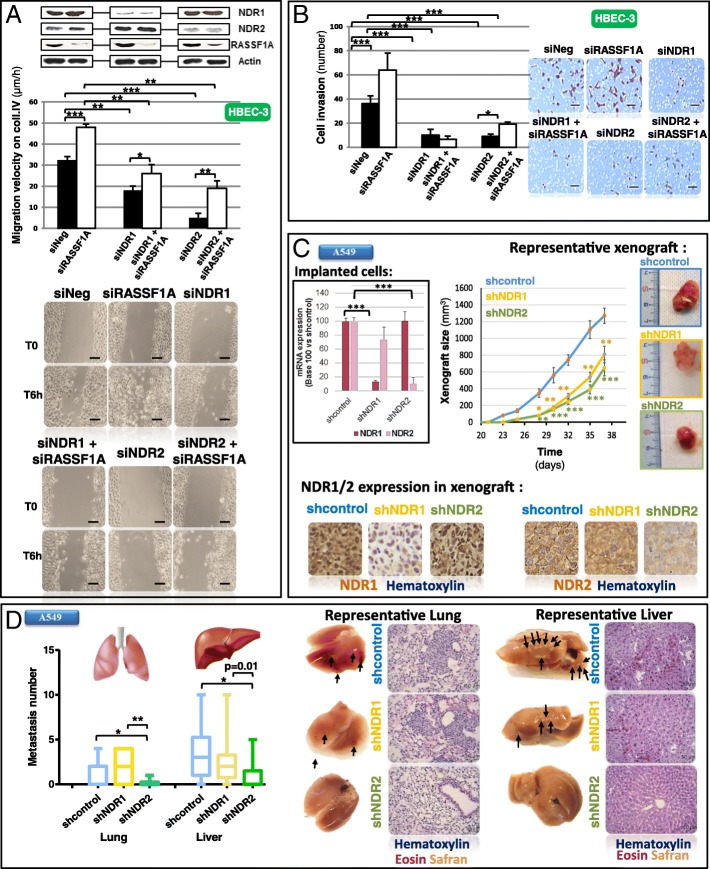
NDR depletion abolishes mobility and metastasis properties in HBEC with RASSF1A depletion. HBEC-3 were transfected with non-silencing siRNA (siNeg), siRASSF1A and/or with siNDR1 or siNDR2. Experiences were performed 48 h after transfection. A549 cells were transfected with shcontrol, shNDR1 or shNDR2. **a** Wound healing assay of HBEC-3 cells on collagen IV coating were performed 48 h after transfection. Scale bar represents 100 μm. **b** Invasion capacity of HBEC-3 cells on BioCoat Matrigel Invasion Chamber. Relative invasion normalized to that of the cells transfected with siNeg. Scale bar represents 80 μm. **c**-**d** ShNDR1 or shNDR2-infected A549 cells suspension were injected subcutaneously in SCID^−/−^ Beige mice. **c** Xenograft tumor size [length (L)/width (l)/thickness (e)]. NDR1 and NDR2 mRNA levels of the injected cells, representative xenograft and NDR1 or NDR2 expression assayed by immunohistochemistry on xenografts are presented respectively on the left of, on the right of and below the xenografts growth curves. **d** Quantification of lung and liver microscopic nodules metastases for A549 cells expressing shNDR1, shNDR2 or shcontrol. Excised mice lung and liver as histologic photographs of the lung and liver metastases after injection with shNDR1, shNDR2 or shcontrol are presented below the quantification. Error bars indicate the standard error of the mean (SEM) (*n* ≥ 3). *P < 0.05, **P < 0.01 and ***P < 0.001, using an ANOVA test followed by Dunnett test


Forty-eight hours after silencing RASSF1A or NDR1/2 in HBEC cells using siRNAs (Additional file 1: Table S2), we performed wound healing (Fig. 1a) and invasion (Fig. 1b) assays in the presence of mitomycin C, to inhibit the contribution of cell division in wound repair. The increase of migration and invasion induced by RASSF1A loss in HBEC-3 cells ([3], Fig. 1a and b) was inhibited by NDR1/2 depletion (Fig. 1a and b) without leading to cell death (cytochrome c release: Additional file 2: Figure S1A, cell viability: Additional file 2: Figure S1B). NDR1/2 silencing also decreased the migratory and invasive properties in RASSF1A-null HBEC as illustrated for H1299 (Additional file 2: Figure S2A/Figure S2B), A549 (Additional file 2: Figure S2A/Figure S2B) and H1650 lung cancer cells (Additional file 2: Figure S2A/Figure S2B, and Additional file 3: Movie S1/ Additional file 4: Movie S2 for siNeg and siNDR2 respectively) again, without leading to cell death (Additional file 2: Figure S2C, except for NDR2 silencing in H1650 cells). To ensure that the observed effects are only related to the extinction of NDR1 or NDR2 and not to off-target effects, we have transfected HBEC-3 cells with siNDR1 and NDR1 plasmid (plsNDR1) or with siNDR2 and NDR2 plasmid (plsNDR2), to restore a basal expression of NDR1 or 2 in cells of which endogenous NDR1 or 2 was silenced (Additional file 2: Figure S3A), before reevaluating 2D (wound healing, (Additional file 2: Figure S3B)) and 3D migration (Additional file 2: Figure S3C). With these experiments, we confirm that siNDR1 or siNDR2 alone actually decreases 2D migration velocity (Additional file 2: Figure S3B) or 3D migration (Additional file 2: Figure S3C), and that these effects are exclusively due to NDR1 or NDR2 silencing, since cells rescued for their expression of NDR1 or NDR2 (transfected by siNDR1 + plasmideNDR1 or siNDR2 + plasmideNDR2), moved as did control cells (siNeg) (Additional file 2: Figure S3B and Figure S3C).

Additional file 3:siNeg transfected H1650 cells migration on Collagen IV. (WMV 2216 kb)



Additional file 4:siNDR2 transfected H1650 cells migration on Collagen IV. (WMV 1513 kb)


We next infected RASSF1A-null A549 or H1299 cells with lentivirus expressing shRNAs targeting NDR1 or NDR2 (A549: Fig. 1c, H1299: Additional file 2: Figure S2D). In SCID−/− Beige mice, tumors formed by shNDR1- or shNDR2 A549 cells emerged later than tumors formed by shcontrol-A549 cells and grew more slowly (Fig. 1c). In H1299 cells, carrying a p53 homozygous deletion and a RASSF1A-gene methylation [12], NDR1 deletion did not affect xenograft growth compared to controls (Additional file 2: Figure S2D) while tumors formed by NDR2-depleted H1299 cells were smaller in size than controls (Additional file 2: Figure S2D). NDR2-depleted cancer cells formed less lung and liver metastatic foci than sh-control or shNDR1-depleted cells (A549: Fig. 1d, H1299: Additional file 2: Figure S2E). Immunohistochemistry and Western blot analysis confirmed that shNDR1 or shNDR2 infected primary tumors actually exhibited decreased NDR1 or NDR2 expression (A549: Fig. 1c and Additional file 2: Figure S4A, H1299: Additional file 2: Figure S2D and Additional file 2: Figure S4B).

Migratory and metastatic phenotypes induced by RASSF1A loss in HBEC could be thus reverted by NDR1/2 silencing.

### NDR-kinases depletion partly reverts EMT induced by RASSF1A loss

**Fig. 2:**
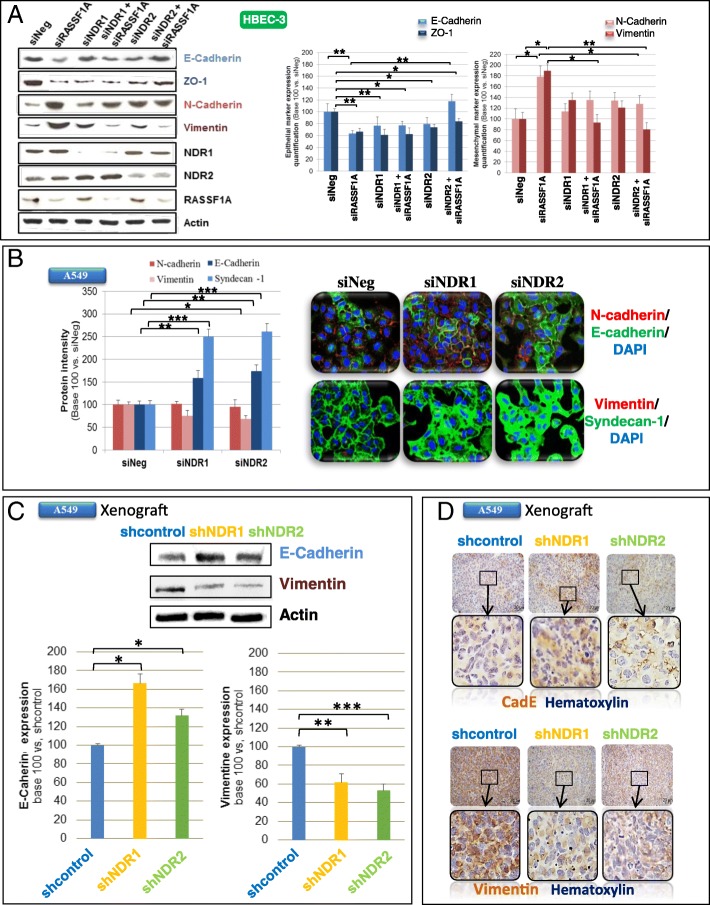
NDR depletion abolishes EMT induced by RASSF1A silencing in HBEC cells. HBEC-3 (**a**) or A549 (**b**) cells were transfected with siNeg, siRASSF1A and/or with siNDR1 or siNDR2. **c**-**d** Xenograft obtained from shcontrol, shNDR1 or NDR2 A549 cells. Quantification of E-Cadherin, syndecan-1, ZO-1, vimentin and/or N-cadherin by western blot (**a**, **c**), Immunofluorescence (**b**) or immunohistochemistry (**d**). Error bars indicate the SEM (*n* ≥ 3). *P < 0.05, **P < 0.01 and ***P < 0.001, using an ANOVA test followed by Dunnett’s test

RASSF1A depletion increased HBEC motility in part by inducing EMT [3]. We tested whether NDR1/2 silencing could revert EMT phenotype. NDR1 as NDR2 depletion abolished mesenchymal marker expression in RASSF1A-depleted HBEC-3 cells (Fig. 2a, bottom histogram). NDR2 silencing restored E-Cadherin, but not ZO-1 expression, upon RASSF1A knockdown (Fig. 2a, upper histogram). The expression of epithelial and mesenchymal markers was also altered by NDR1/2 manipulations in two other lung cancer cells null for RASSF1A (H1299: Additional file 2: Figure S5A, A549: Fig. 2b). NDR1 as NDR2 depletion increased epithelial marker (Fig. 2b, cf. E-Cadherin and Syndecan-1) and decreased Vimentin (Fig. 2b) expression in RASSF1A-null A549 cells. Finally, in RASSF1A-null H1299 cells, NDR1 as NDR2 depletion increased Syndecan-1 (Additional file 2: Figure S5A) and decreased Vimentin (Additional file 2: Figure S5A) expression.

In HBEC xenografts, upon NDR2 depletion, E-cadherin is also report to be slightly increased (A549 xenografts: Fig. 2c, H1299 xenografts: Additional file 2: Figure S5B) and restored at the membrane (A549 xenografts: Fig. 2d, H1299 xenografts: Additional file 2: Figure S5C). Conversely, vimentin expression is decreased by NDR1 or NDR2 knockdown in A549 (Fig. 2c and d) or H1299 (Additional file 2: Figure S5B and Additional file 2: Figure S5C) xenografts when compared to shControl xenografts.

NDR1/2 knockdown could thus partly prevent EMT upon RASSF1A silencing in HBEC.

### NDR-kinases depletion impairs YAP activation in RASSF1A-depleted HBEC

**Fig. 3:**
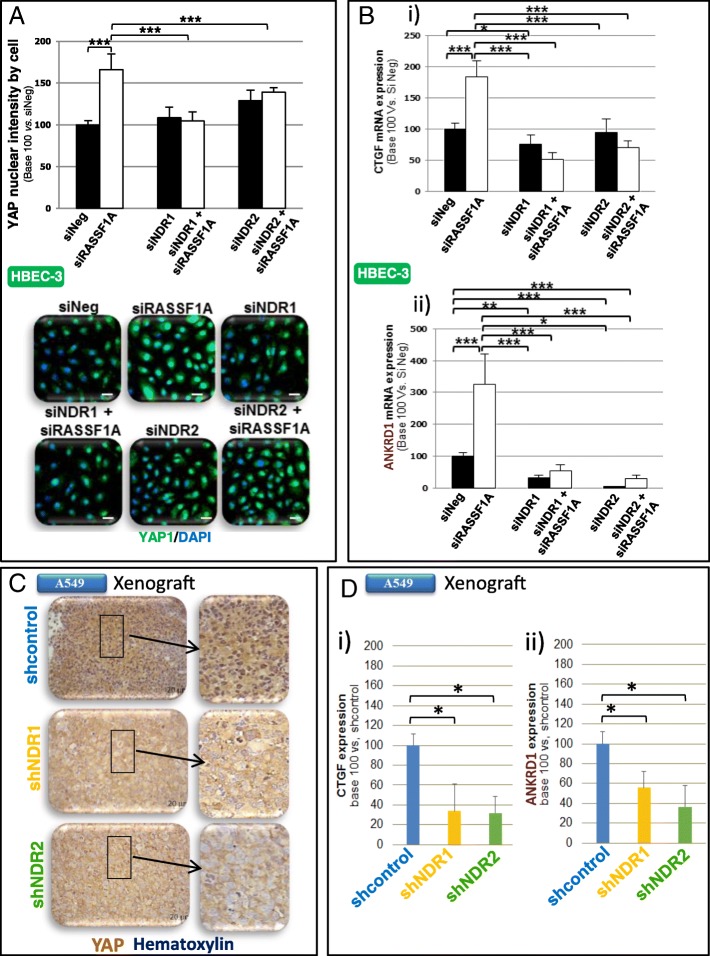
NDR depletion abolishes YAP activation induced by RASSF1A silencing in HBEC cells. **a**-**b** HBEC-3 cells were transfected with siNeg, siRASSF1A and/or with siNDR1 or siNDR2. **c**-**d** Xenograft obtained after subcutaneous injection of shcontrol, shNDR1 or NDR2 H1299 cells. Nuclear YAP quantification by immunofluorescence (**a**) or by immunohistochemistry (**c**). **b**, **d** Quantification of CTGF (Bi, Di) & ANKRD1 (Bii, Dii) mRNA using actin as an internal control in HBEC-3 cells. Error bars indicate the SEM (*n* ≥ 3). *P < 0.05, **P < 0.01 and ***P < 0.001, using an ANOVA test followed by Dunnett’s test

Increased migration of RASSF1A-depleted HBEC-3 cells being YAP-dependent, ([3], Fig. 3a), we wondered whether NDR1/2 depletion could revert YAP activity upon RASSF1A loss. We found that NDR1 or NDR2 depletion did prevent YAP nuclear localization in RASSF1A-depleted (HBEC-3, Fig. 3a) or -null (A549 and H1650, Additional file 2: Figure S6A) cells. NDR1 or NDR2 silencing also diminished CTGF and ANKRD1 mRNA (Fig. 3 Bi-ii and Additional file 2: Figure S6Bi-ii), two transcriptional targets of YAP [13]. Nuclear YAP intensity in RASSF1A-null xenografts depleted for NDR1 or NDR2 was reduced compared to shControl xenografts (H1299: Additional file 2: Figure S6C, A549: Fig. 3c), and led to partly YAP inactivation as shown by the diminished CTGF and ANKRD1 mRNA in A549 xenografts depleted for NDR1 or NDR2 compared to shControl (Fig. 3 Di-ii).

Thus, in RASSF1A-depleted HBEC, NDR1/2 could get activated and lead to the YAP activation with a subsequent increase of motility.

### NDR2 contributes to the regulation of RhoB activity

**Fig. 4:**
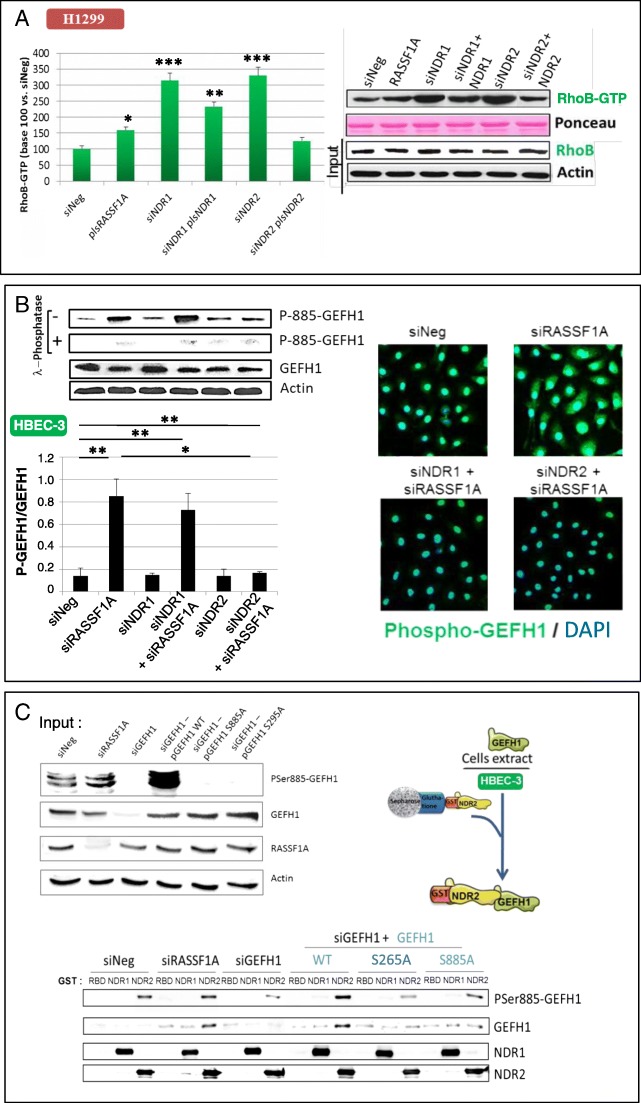
NDR2 interacts and phosphorylates GEF-H1 in HBEC. HBEC-3 or H1299 cells were transfected with siNeg and/or siRASSF1A, siNDR1, siNDR2, siGEF-H1, pcDNA3-NDR1, pcDNA3-NDR2 or pcB6-GEF-H1. **a** GST-RBD pull-down assay in H1299 cells. **b** Ser885 phosphorylation from GEF-H1 assayed by western blot following λ-phosphatase pre-treatment of the total protein extract or not and normalized with total GEF-H1 expression in HBEC-3 cells. **c** GST-NDR1 or -NDR2 pull-down assay using siRNA & GEF-H1 plasmid as controls. NDR2 phosphorylation on Ser265-GEFGH1 link with phosphorylation on Ser-885-GEF-H1A was assayed. NDR2 activity on HBEC-3 cells extracts previously transfected with pcB6-GEF-H1 wild type, mutated on Ser265 (S265A), or on Ser885 (S885A) was assayed by quantifying ser885-GEF-H1 phosphorylation status by western blot following normalization by total GEF-H1 expression. Error bars indicate the SEM (*n* ≥ 3). *P < 0.05, **P < 0.01 and ***P < 0.001, using an ANOVA test followed by Dunnett’s test

The RhoB GTPase playing an important role in the migration downstream of RASSF1A loss [3], we tested whether NDR1/2 contribute to the RhoB regulation upon RASSF1A loss. By forcing re-expression of RASSF1A in H1299 cells, we observed an increase in the activated (GTP-bound) form of RhoB (Fig. 4a). Depletion of NDR1/2 increased the levels of the active GTP-bound RhoB form, while the restoration NDR1 or NDR2 expression decreased the GTP-bound RhoB (Fig. 4a). We further confirmed that depletion of RASSF1A decreased the levels of active form RhoB ([3], Additional file 2: Figure S7A) and reported that RhoB activity was restored in RASSF1A-depleted HBEC-3 cells upon NDR2 silencing (Additional file 2: Figure S7A) but not upon NDR1 silencing (Additional file 2: Figure S7A). NDR2 depletion can thus restore RhoB activation in RASSF1A null HBECs.

### NDR2 interacts with GEF-H1 resulting in S885-GEF-H1 hyper-phosphorylation and subsequent GEF-H1 inactivation

Considering that RhoB is regulated by NDR2 (Fig. 4a and Additional file 2: Figure S7A) and GEF-H1 [3, 14], we tested whether NDR1/2 are involved in GEF-H1 regulation. In line with our previous finding [3], we showed that NDR2 but not NDR1 silencing decreased GEF-H1 phosphorylation upon RASSF1A loss (Fig. 4b). The specificity of the phospho-Ser885-GEF-H1 antibody was further supported by λ-phosphatase pretreatment of the protein extract, which actually abolished the phosphorylation band revealed in GEF-H1 (Fig. 4b). NDR2 could thus regulate the GEF-H1 activity and then, the regulation of RhoB by GEF-H1.

**Fig. 5:**
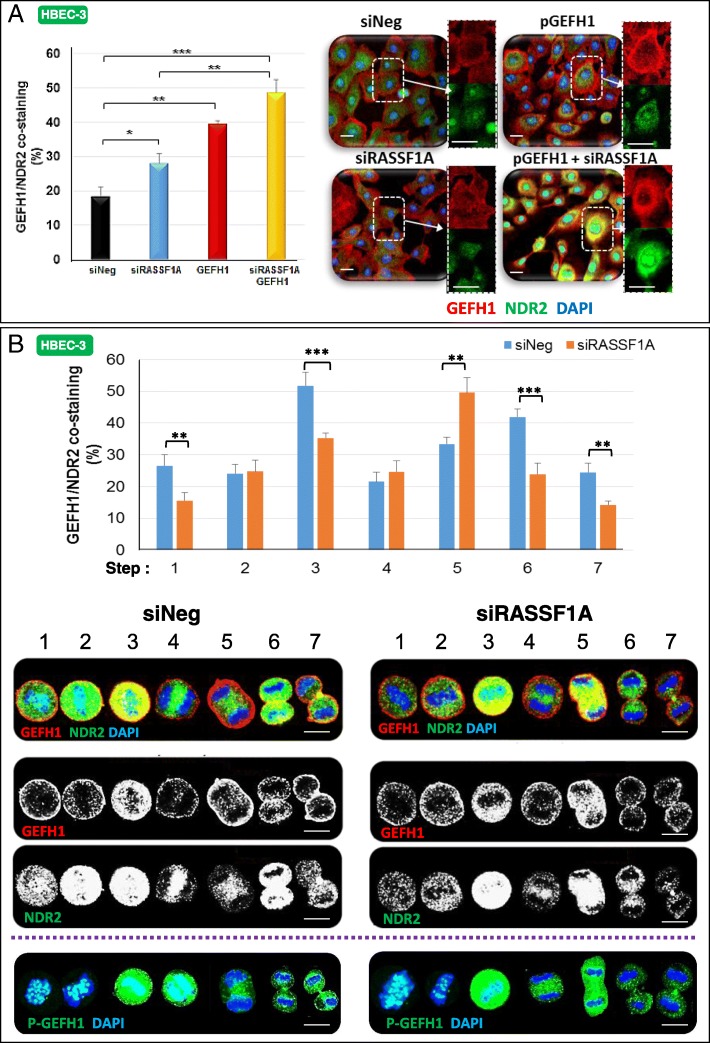
NDR2 and GEF-H1 are co-stained in HBEC-3 cells during both interphase and cell division. HBEC-3 cells were transfected with siNeg or siRASSF1A. **a** GEF-H1 and NDR2 co-staining assayed by immunofluorescence in HBEC-3 cells during interphase. **b** Representative images are shown for NDR2 and GEF-H1 during mitosis. Localization was identified by immunofluorescence and confocal microscopy. Costaining was evaluated by ImageJ software. HBEC-3 cells were also stained with DAPI for DNA and PSer885GEF-H1 during mitosis. Error bars indicate the SEM (*n* ≥ 3). *P < 0.05, **P < 0.01 and ***P < 0.001, using an ANOVA test followed by Dunnett’s test

Observing a stronger GEF-H1/NDR2 co-staining in RASSF1A-depleted HBEC-3 cells than in controls, as well as a co-staining in RASSF1A-depleted HBEC-3 cells expressing an exogenous wild-type form of GEF-H1 (Fig. 5a), we tested whether GEF-H1 can interact with NDR2. We failed to detect an NDR2/GEFH1 interaction by co-immunoprecipitation with available antibodies (not shown), and tested whether this interaction could be favored by another partner. Indeed, syndecan-1 (SDC1), was previously shown by others to interact with GEF-H1 [15] and influence Rho activation [16], while we also reported SDC1 decreased upon RASSF1A loss [3]. Actually, in SDC1 immunoprecipitates from HBEC-3 cells extracts, we were able to detect both NDR2 and GEFH1, while none of them was detected upon SDC-1 silencing (Additional file 2: Figure S7B). Thus, an in vivo interaction between NDR2 and GEF-H1 does occur needing SDC1 mediation.

Using recombinant GST-NDR2, we also pulled-down endogenous GEF-H1 from HBEC-3 cell extracts (Fig. 4c, GST-assay while, endogenous GEF-H1 was not pull-down with GST-NDR1 or upon GEF-H1 knockdown (Fig. 4c). We expressed RNAi-resistant GEF-H1 versions in GEF-H1-depleted cells, and consequently tested their binding to GST-NDR2 (Fig. 4c). We compared wild-type GEF-H1 (pls WT-GEF-H1) with two phosphor-acceptor mutants termed GEF-H1 S265A and GEF-H1 S885A (Additional file 2: Figure S8) since S265 is included in the putative NDR phosphorylation motif HXRXXS/T [17] and S885 contributes to GEF-H1 activity [18]. We detected a strong Ser885 phosphorylation of GEF-H1 in total protein cell extract from WT-GEF-H1-transfected HBEC-3 cells, but not in extracts from GEF-H1-S885A or GEF-H1-S265A-transfected HBEC-3 cells (Fig. 4c, input, top lane). Finally, exogenous GST-NDR2 kinase induced strong GEF-H1 Ser885 phosphorylation on exogenously expressed wild-type GEFH-1 retained on beads, while NDR2 beads only retained a low amount of phosphoSer885 GEF-H1 on exogenously expressed S885A, and more interestingly S265A GEF-H1 mutants (Fig. 4c, GST assay). All these data suggest a link between the phosphorylations of Ser265-GEF-H1 and Ser885-GEF-H1.

### RASSF1A depletion delays abscission and alters cytokinesis in bronchial cells lines

**Fig. 6:**
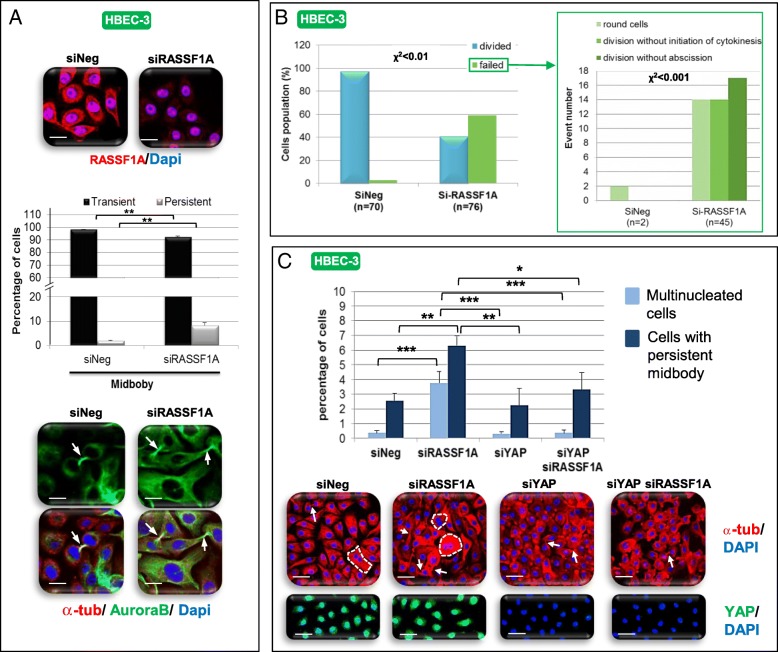
RASSF1A depletion induces YAP-dependent cytokinesis defect. HBEC-3 cells were transfected with si-RASSF1A, siYAP and/or si-Neg. Cells were stained with anti-RASSF1A, anti-tubulin and/or anti-AuroraB antibodies and DAPI. Persistent midbody was quantified (**a**) as the number of cells failing to divide following cytokinesis defect by scoring > 100 cells, imaged at 2 min intervals when rounded up (**b**). **c** Percentage of HBEC-3 cells multinucleated and/or with persistent midbody evaluated following RASSF1A and YAP silencing and immunostaining of the alpha-tubulin with DAPI for the nucleus. **a**, **c** Scale bar represents 50 μm. Error bars indicate the SEM (*n* ≥ 3). *P < 0.05, **P < 0.01 and ***P < 0.001, using an ANOVA test followed by Dunnett’s test. **b** Correlation between RASSF1A presence and events was test using a Chi2 test

RASSF1A [19, 20], NDR1/2 kinases [21], GEF-H1 [10, 22] and YAP [23] controlled mitosis, but whether these proteins act together is not understood. We first characterized the mitotic phenotypes associated with RASSF1A loss in HBECs. We detected no alteration in the equatorial plan definition or the equatorial structure processing in RASSF1A-depleted HBEC, as evidenced by normal staining of the main protagonists involved in such steps: MKLP1 (Additional file 2: Figure S9A), PRC1 (Additional file 2: Figure S9B) RhoA (Additional file 2: Figure S9C), Rac1 (Additional file 2: Figure S9D), or Ect2 (Additional file 2: Figure S9E). Conversely, RASSF1A inactivation increased chromosomes misalignment (Additional file 2: Figure S10A) and lagging (Additional file 2: Figure S10B) in HBEC-3 as in HBEC-3-RasV12 cells (Additional file 2: Figure S10C). RASSF1A inactivation also increased midbody persistence in HBEC-3 (Fig. 6a) as in HBEC-3-RasV12 cells (Additional file 2: Figure S10D) as evidenced by α-tubulin and Aurora B co-staining [24] (Fig. 6a, Additional file 2: Figure S10D) and by the modification of expression of Anillin (Additional file 2: Figure S10F), Aurora B (Additional file 2: Figure S10G) and Citron kinase (Additional file 2: Figure S10H). RASSF1A loss delayed the transition from the onset of furrowing to completion of abscission (Additional file 2: Figure S10I, Additional file 6: Movie S4/ Additional file 7: Movie S5) and increased the number of cells with failing mitosis evidenced by increased numbers of ***i)*** round cells never entering into mitosis (Fig. 6b, Additional file 8: MovieS6), ***ii)*** cells never initiating cytokinesis (Fig. 6b, Additional file 9: MovieS7), or ***iii)*** cells never terminating abscission and exhibiting broad cytoplasmic bridges interconnecting daughter cells (Fig. 6b, Additional file 10: MovieS8) and ***iv)*** of bi- or multi-nucleated HBEC-3 (Additional file 2: Figure S10 J) or HBEC-3-RasV12 cells (Additional file 2: Figure S10Q), with independent initiation of mitosis for nuclei from a same HBEC-3 cell (shown by confocal acquisition of siRASSF1A transfected cells, Additional file 5: MovieS3). Supporting the midbody abscission defect we suspected, we reported accumulation of Spastin and Fidgetin, two enzymes involved in midbody cut (Additional file 2: Figure S11A), and alterations in the content of Rab11 (increased) and Syntaxin16 (decreased) (Additional file 2: Figure S11B), two crucial proteins for intracellular traffic and mitosis [25, 26]. Thereby, RASSF1A depletion affected cytokinesis beyond the only step of the midbody formation described by others [20].

### Cytokinesis disorders induced by RASSF1A depletion are YAP-dependent

We tested the contribution of YAP on the cytokinesis phenotypes associated with RASSF1A loss. YAP depletion alone did no affect the number of multinucleated cells or persistent midbodies compared to controls (Fig. 6c) but significantly decreased the number of multinucleated cells and persistent midbody in RASSF1A-depleted HBEC-3 (Fig. 6c). Thus, the cytokinesis disorders induced by RASSF1A loss could be dependent of YAP in HEC.

### GEF-H1 and NDR2 co-staining is divergent upon RASSF1A loss during mitosis

We wondered whether NDR2-associated GEF-H1 inactivation in RASSF1A-depleted cells could be responsible for the cytokinesis defects observed upon RASSF1A loss. In wild-type HBEC-3 cells, GEF-H1 and NDR2 co-staining was mainly observed at the early prophase, during the establishment of the equatorial plane, at the end of telophase and during abscission (at the cleavage point) (Fig. 5b) suggesting an involvement of NDR2 in the regulation of GEF-H1 activity during these mitotic steps. In RASSF1A-depleted HBEC-3 cells, NDR2/GEF-H1 co-staining features were considerably different at (Fig. 5b): ***i)*** the early prophase (the sub-cortical co-staining signal observed in control cells were lower) ***ii)*** the equatorial plane step (co-staining was fainter than in controls), ***iii)*** the contractile ring assembly (co-staining was stronger in RASSF1A-depleted cells) and ***iv)*** the abscission step (with a decreased signal at the midbody). NDR2 and GEF-H1 do not localize identically in RASSF1A-depleted or RASSF1A wild type HBEC, suggesting that mitosis alteration in RASSF1A depleted-cells could be transmitted by altered NDR2 and GEF-H1 localization/activation.

### GEF-H1 depletion mimics cytokinesis defects induced by RASSF1A loss

Testing whether the inactivation of GEF-H1 could explain the cytokinesis disorders observed upon RASSF1A loss, we depleted GEF-H1 in HBEC-3 cells (Additional file 2: Figure S12A). Single depletion of RASSF1A elevated the number of multinucleated cells compared to controls (Additional file 2: Figure S12A) while GEF-H1 silencing increased the proportion of binucleated cells (Additional file 2: Figure S12B) without affecting the number of persistent midbodies (Additional file 2: Figure S12C). Co-depletion of RASSF1A and GEF-H1 did not increase the number of multinucleated cells (Additional file 2: Figure S12A) but increased the proportion of attached daughter cells (Additional file 2: Figure S12B). GEF-H1 inactivation could thus lead to cytokinesis disorders in HBEC.

### GEF-H1 overexpression, or NDR2 knockdown restores cytokinesis in RASSF1A-depleted cells

**Fig. 7:**
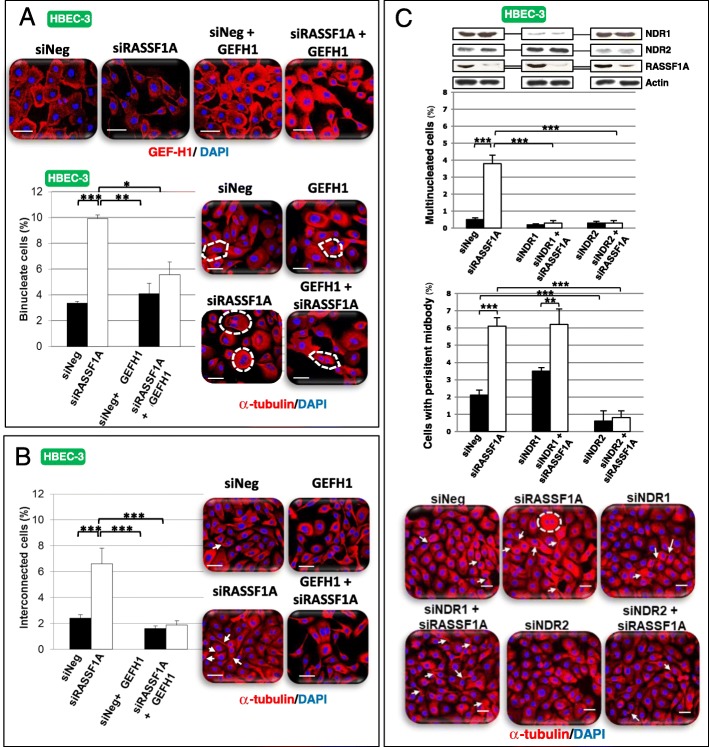
GEF-H1 overexpression as NDR depletion restores cytokinesis of RASSF1A depleted human bronchial cells. HBEC-3 (**a**-**c**) were transfected with siNeg, siRASSF1A and/or with siNDR1, siNDR2 or pcB6-GEF-H1 (**a**). The number of binucleate (**a**, **c**) and interconnected cells (**b**, **c**) were counted after alpha-tubulin and DAPI staining from cells over-expressing or not GEF-H1 (**a**) or silenced for NDR kinases (**c**) in HBEC-3. These numbers are expressed as a percentage in control and siRNA-transfected cells. Error bars indicate the SEM (*n* ≥ 3). *P < 0.05, **P < 0.01 and ***P < 0.001, using an ANOVA test followed by Dunnett’s test

To further examine the mitotic link between GEF-H1 and NDR2 upon RASSF1A loss, we overexpressed wild-type GEF-H1 or silenced NDR2 in RASSF1A-depleted HBEC-3 cells. GEF-H1 overexpression (Fig. 7a) restored cytokinesis in RASSF1A-depleted cells as judged by the number of bi-nucleated cells (Fig. 7a) or cells with persistent midbodies (Fig. 7b) compared with controls cells. Similarly, NDR1 or NDR2 depletion decreased the rate of multinucleated cells upon RASSF1A loss (Fig. 7c), however only NDR2 silencing suppressed the formation of persistent midbodies (Fig. 7 c). Alike, NDR2, but not NDR1 depletion in RASSF1A-null H1299 cells decreased the number of multinucleated cells and persistent midbody (Additional file 2: Figure S12C). Thus, both GEF-H1 and NDR2 could function as mediators of cytokinesis failures upon RASSF1A loss.

### Low RASSF1A, low RhoB-GEF-H1 or high NDR2 kinase mRNA cell content predicted worse overall survival of lung cancer patients

Consistently with a molecular machinery where RASSF1A, NDR2, GEF-H1 and RhoB work in concert, analysis of survival of resected early lung cancer patients from The Cancer Genome Atlas cohort, showed that low mRNA expression of RASSF1A (Additional file 2: Figure S13A), RhoB (Additional file 2: Figure S13B), and GEF-H1 (Additional file 2: Figure S13C) predict worse overall survival in NSCLC patients as high expression of NDR2 mRNA (Additional file 2: Figure S13C).

## Discussion

We recently reported that RASSF1A acts both as a tumor and metastasis gene suppressor in patients with early stage non-small cell lung cancer (NSCLC) [1, 3], RASSF1A both restricting activation of YAP, one of the terminal target of the Hippo pathway, and stimulating activation of RhoB, a small anti-migratory GTPase protein, via GEF-H1, its GDP/GTP exchange factor [3]. Here, we report that the NDR2 kinase is required for YAP activation and RhoB/GEF-H1 inactivation in RASSF1A-depleted human bronchial cells, consequently supporting the pro-invasive and cytokinesis disorders upon RASSF1A loss.

Our report of a pro-oncogenic role for a NDR kinase, in a context where RASSF1A is lost, is rather unexpected, taking into account for a previously reported role as a YAP kinase, contributing to YAP cytoplasmic sequestration, but in line with recent studies in Ras-transformed human cells [27, 28]. Indeed, in Ras transformed HEK-HT cells, NDR1 knockdown was shown to impair anchorage-independent soft agar cell growth and in vivo xenograft growth [27]. In addition, that NDR1/2 kinases are involved in the control of these movements is reminiscent of a comparable function assigned to their close homologs, the LATS1/2 kinases [29, 30]. Mechanistically, such action could involve NDR2 regulation of β1 integrin [31], which is required for invasive cell capabilities [32] and known to regulate nuclear location of YAP1 [33]. In our model, we previously reported a nuclear increase of YAP in RASSF1A-depleted cells, while we show here, that NDR2 activity is required for such YAP activation (Fig. 3, Additional file 2: Figure S6). However, to date, there is no report of a link between the NDR kinases activities and extracellular matrix remodeling, so an alternative hypothesis explaining how NDR kinase influences cell motility could involve its control on cell adhesion and/or cytoskeleton remodeling, NDR kinase pathway being previously reported to coordinate cell cycle dependent actin rearrangements [34].

That NDR kinases control cell motility is also consistent with the contribution of NDR kinases to EMT/MET processes (Fig. 2, Additional file 2: Figure S5), previously suggested by the report of NDR kinases influence on cell differentiation during organogenesis [35] and their ability to inhibit TGFβ [36], a cytokine leading squamous differentiation of non-transformed HBEC [37]. In our model, the control of NDR on the mesenchymal phenotype appears related to the control that NDR kinases exert on YAP. Indeed, we had reported that the EMT induced by RASSF1A silencing in HBEC was correlated with the abnormal activation of YAP in these cells [3]. Here, we show that in the absence of NDR, the EMT of RASSF1A-depleted HBEC is partially reverted, which is correlated with YAP inactivation in HBEC lacking both RASSF1A and NDR kinases.

We also report here, that NDR loss can revert migratory and cytokinetic abnormalities of RASSF1A-depleted cells by preventing YAP activation (Fig. 3, Additional file 2: Figure S6). NDR kinases may therefore up- or down-regulate YAP activity, depending on the cellular context, which could explain these kinases may behave as oncogenes or tumor suppressor genes [7]. YAP is indeed able to exert pro-oncogenic action by reprogramming cells behavior [38], or anti-oncogenic functions, by inhibiting tumors occurrence in mice [39]. An oncogenic function of NDR2 is supported by reports, showing that NDR2 supports MYC protein stability [40] and thus contributes to oncogenic Ras transformation [28]. In addition, high level of NDR1 [41] or NDR2 [42] mRNAs were found in non-small cell lung cancer. Our work originally shows that a NDR kinase contributes to oncogenic functions in a lung cancer model involving cancer cells carrying Ras (A549 cells with Ras G12S) or p53 mutations (p53-deleted H1299 cells).

NDR kinases leading to the nuclear exclusion of YAP [7], it was surprising here that the depletion of NDR kinases decreased YAP nuclear levels and activity in cells lacking of RASSF1A. We suggest that in RASSF1A-depleted HBECs, the nuclear localization of YAP is possibly not linked to the canonical Hippo pathway, but rather connected to the RhoB inactivation [3], such being the situation when mechano-transduction mobilizes YAP1 [43]. Actually, we found that NDR depletion reverts the inhibition of GEF-H1/RhoB in RASSF1A-depleted cells (Fig. 4, Additional file 2: Figure S7, Fig. 5). This finding is fully consistent with our previous work demonstrating that the nuclear localization of YAP is caused by the inactivation of RhoB upon loss of RASSF1A [3]. Our data showing that NDR kinases are indeed at the origin of the inactivation of RhoB and activation of YAP, also suggest that NDR could be involved in the mechano-transduction regulation.

The NDR2 kinase could therefore be one of the kinases responsible for the inactivation of GEF-H1, and the subsequent RhoB inactivation in RASSF1A depleted cells. In support of this hypothesis we observed that (1) NDR2 depletion decreased Ser885-GEF-H1 phosphorylation (i.e. NDR2 depletion increased the levels of inactive GEF-H1) in RASSF1A-depleted cells (Fig. 4b), (2) NDR2 depletion increased the levels of GTP-bound, hence activated RhoB (Fig. 4a, Additional file 2: Figure S7A), (3) NDR2 displayed increased co-staining with phosphorylated Ser885-GEF-H1 in RASSF1A depleted-HBEC (Fig. 5a), (4) using exogenous proteins, NDR2 can interact with GEF-H1 in a fashion dependent on Ser265 and Ser885 (Fig. 4c), and (5) using co-immunoprecipitation of SDC1-containing complexes, we were able to show an in vivo association of GEF-H1 and NDR2 (Additional file 2: Figure S7B). Collectively, these findings actually suggest that NDR2 can directly interact with, and inhibit GEF-H1. Possibly, NDR2-induced GEF-H1 phosphorylation on Ser265, might in turn lead to increased phosphorylation of GEF-H1 Ser885 by another kinase, consequently resulting in the loss of GEF-H1 activity (Fig. 4, Additional file 2: Figure S7, Fig. 5). However, this model remains speculative at the moment, requiring intensive structure focused studies in the future. Nevertheless, our hypothesis is further supported by recent studies showing that GEF-H1 can actually be phosphorylated on Ser265 [44] and that GEFs are frequently inactivated by successive phosphorylation events [45].

The link we established between the RhoB/GEF-H1/NDR2/YAP and RASSF1A signaling in the context of EMT and migration led us to explore another process deregulated in RASSF1A depleted HBEC, namely mitosis/cytokinesis. We provide evidence that the deregulation of one member of this interactome is sufficient to disrupt cytokinesis in RASSF1A-knockdown cells, a feature consistent with ***i)*** the RASSF1A localization with the microtubules at contractile ring and midbody [46, 47], ***ii)*** the YAP localization to the midbody and spindle [23], ***iii)*** the GEF-H1 localization at the tips of cortical microtubules and the midbody [10], ***iv)*** the lower co-staining of GEF-H1 and NDR2 at early prophase, at equatorial plane positioning and during midbody formation, when compared to RASSF1A wild-type cells (Fig. 6). Such result sustains the role of RASSF1A/NDR2/GEF-H1/RhoB axis in cytokinesis in addition to its role in cell migration that we also support here.

## Conclusion

**Fig. 8:**
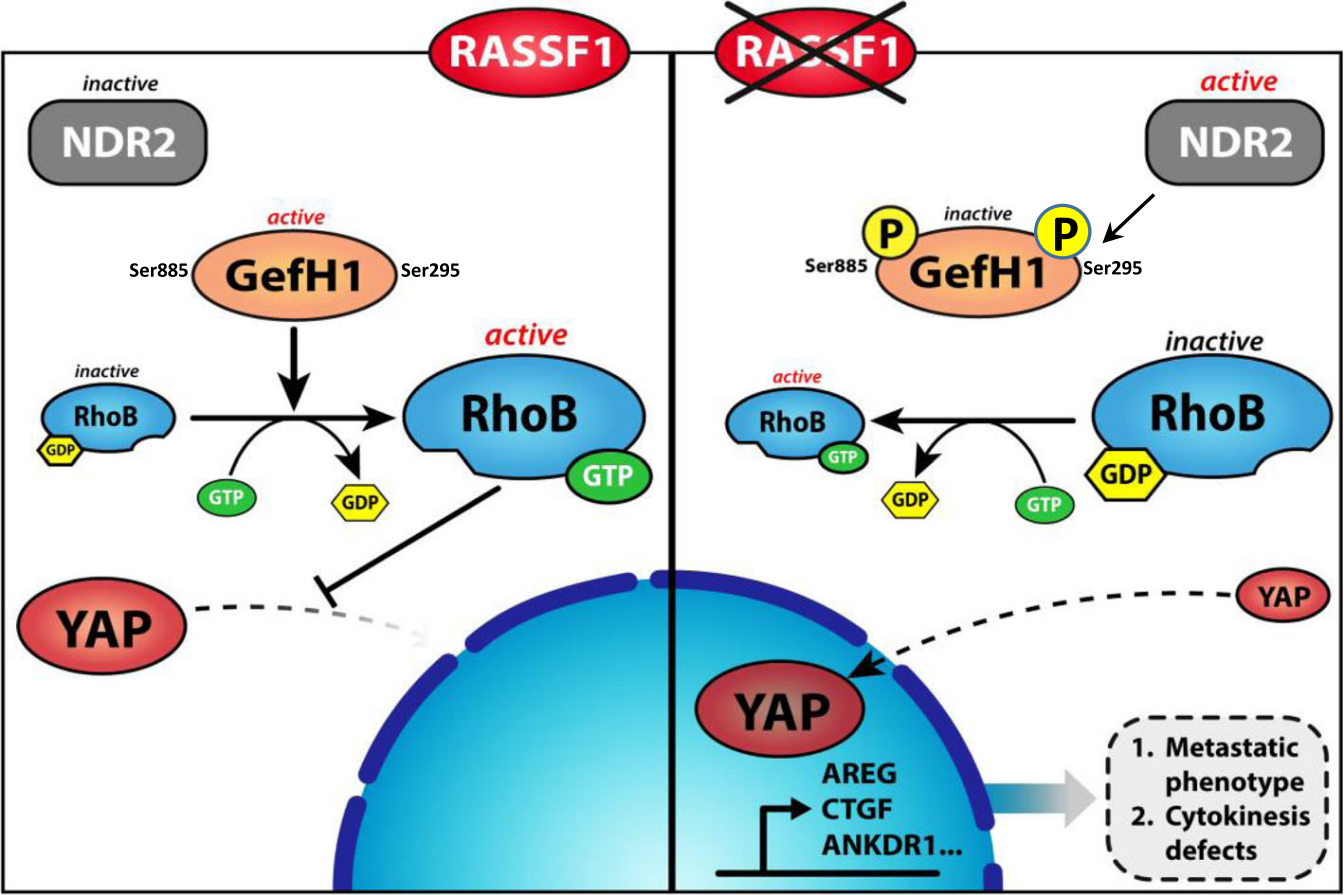
A proposal model for RASSF1A regulation of the NDR2/GEFH-1/RhoB/YAP axis

We propose the following model: in healthy bronchial lung cells, RASSF1A inhibits NDR2, leaving rhoGEF-H1 active and leading to RhoB activation, which can exert in turn its anti-metastatic activity (Fig. 8). Thus, NDR2 kinase appears to be a potential therapeutic target in patients with lung cancer and loss of RASSF1A expression, who account for more than 30% of all lung cancer patients.

## Additional files

Additional file 1: **Table S1.** Characteristics of the cell lines used in the study. **Table S2.** Primers and siRNA sequences used in this work. **Table S3.** Antibodies used in this work. (DOCX 27 kb)

Additional file 2: **Figure S1.** NDR1 or NDR2 depletion abolishes mobility and metastasis properties in HBEC with RASSF1A depletion without lead to cells death. **Figure S2:** NDR2 depletion abolishes mobility and metastasis properties in HBEC with RASSF1A depletion without lead to cells death. **Figure S3.** NDR1 or NDR2 restores mobility and 3D migration in HBEC with respectively NDR1 or NDR2 depletion. **Figure S4.** NDR1/2 expression in shcontrol, shNDR1 or shNDR2 HBEC. **Figure S5. **NDR1orNDR2depletionpartlyabolishedEMTinRASSF1A-depletedH1299cells. **Figure S6.** NDR2depletionabolishesYAPactivationinRASSF1A-depletedA549orH1650cells. **Figure S7.** TheheparansulfateproteoglycanSyndecan-1(SDC-1)mediatestheinteractionandphosphorylationofGEF-H1byNDR2inHBEC-3cells. **Figure S8.** Sanger analysis of GEF-H1 S265A or S885A mutants. **Figure S9.** RASSF1A is not required for cleavage plan or equatorial structure of HBEC. **Figure S10.** RASSF1Adepletion induces defects on metaphase and anaphase and is required for mid body formationin HBEC cells. **Figure S11.** RASSF1A depletion leads to accumulation of enzymes involved in midbody cut (A) and alterations in the content of proteins crucial for intracellular traffic and mitosis (B) in HBEC cells. **Figure S12.** GEF-H1 silencing mimics cytokinesis failure induced by RASSF1A loss in HBEC-3 cells while NDR2 depletion in RASSF1A-depleted H1299 cells restores proper cytokinesis. **Figure S13.** RASSF1A/RhoB/GEF-H1/NDR2 mRNA impacts on survival from of 681 patients with NSCLC,TCGAcohort. (PDF 2685 kb)

Additional file 5: desynchronized nuclei division in siRASSF1A transfected HBEC-3. (WMV 372 kb)

Additional file 6: siNeg transfected HBEC-3 cytokinesis. (WMV 147 kb)

Additional file 7: siRASSF1A transfected HBEC-3 cytokinesis. (WMV 325 kb)

Additional file 8: Cytokinesis failure of siRASSF1A transfected HBEC-3. (WMV 332 kb)

Additional file 9: Cytokinesis failure of siRASSF1A transfected HBEC-3 bis. (WMV 357 kb)

Additional file 10: Cytokinsesis failure of siRASSF1A transfected HBEC-3 ter. (WMV 319 kb)

## Abbreviation

BrdU: Bromodeoxyuridine
EMT: Epithelial-mesenchymal transition
GEF-H1: Guanine nucleotide exchange factor 1
GST: Glutathione-S-transferase
HBEC: Human bronchial epithelial cells
NSCLC: Non-Small Cell Lung Cancer
RASSF1: Ras-association domain family isoform
RBD: Rhotekin Rho binding domain
RT-PCR: Reverse Transcription-Quantitative real-time-PCR
SDC1: Syndecan-1
