# Supplementary material for: NDR2 kinase contributes to cell invasion and cytokinesis defects induced by the inactivation of RASSF1A tumor-suppressor gene in lung cancer cells
Source: J Exp Clin Cancer Res. 2019 Apr 12;38:158. doi: 10.1186/s13046-019-1145-8 (PMC6461807; doi:10.1186/s13046-019-1145-8)
Supplement: Supplementary file 3 — Table S1. Characteristics of the cell lines used in the study. Table S2. Primers and siRNA sequences used in this work. Table S3. Antibodies used in this work. (DOCX 27 kb) [file 13046_2019_1145_MOESM1_ESM.docx]

**Tables.**

**TableS1. Characteristics of the cell lines used in the study.**

| Origin | Cell lines | Origin/  Disease | *RASSF1* promoter gene | Gene Alteration Profiles |
| --- | --- | --- | --- | --- |
| Lung epithelial | HBEC-3 | immortalized Healthy bronchial epithelial cell | Unmethylated | Inactivation of Rb and p16  (*via* CDK4 constitutive expression)  hTERT constitutive expression |
|  | HBEC-3 RasV12 | immortalized healthy bronchial epithelial cell | Unmethylated | Inactivation of Rb and p16  (*via* CDK4 constitutive expression)  hTERT constitutive expression  Activating mutation of Ras |
|  | A549 | lung adenocarcinoma  (bronchoalveolar) | Methylated (RASSF1A null) | K-Ras G12S activating mutation  CDKN2A homozygous deletion (loss of p16 and p14)  STK11 Q37* Nonsense mutation |
|  | H1299 | lung non-small cell carcinoma  (metastatic lymph node) | Methylated  (RASSF1A null) | p53 homozygous deletion (p53 null expression)  N-Ras pQ61K mutation |
|  | H1650 | adenocarcinoma  (metastatic pleural effusion) | Methylated  (RASSF1A null) | EGFR activation mutation (in-frame deletion exon: 19 pE746_A750EL>REA) |

**TableS2**. Primers and siRNA sequences used in this work.

| Target | siRNA sequence (5’>3’) | Quantity per  10cm² | Primers for qRT-PCR  Forward (F, 5’>3’)  Reverse (R, 5’>3’) |
| --- | --- | --- | --- |
| RASSF1A | **siRNA1* :** GACCUCUGUGGCGACUUCATT | 10 nmol | **F**: ggc gtc gtg cgc aaa ggc c  **R** : ggg tgg ctt ctt gct gga ggg |
|  | **siRNA2 :**  CAAGGACGGUUCUUACACA | 10 nmol |  |
| GEF-H1 | **siRNA1* :**  GAAGGUAGCAGCCGUCUGU | 20 nmol | **F**: aca cgc ttc ctc agc cag ctat ta  **R** : aat tgc tgg aag cgt ttg tct cgg |
|  | **siRNA2 :**  GAAUUAAGAUGGAGUUGCAUU | 20 nmol |  |
| Yap | **siRNA1* :**  UGAGAACAAUGACGACCAA | 10 nmol | **F**: GCC GGA GCC CAA ATC C  **R** : GCA GAG AAG CTG GAG AGG AAT G |
|  | **siRNA2 :**  CCACCAAGCUAGAUAAAGA | 10 nmol |  |
| NDR1 | **siRNA1* :** AAGTAATAGGCAGAGGAGCAT | 25 nmol | **F**: GTG AGG TGC GGC TTG TTC A  **R** : GTC ACG CTC CGC ACG AAT |
|  | **siRNA2 :** AAGAGCAGGTTGGCCACATTC | 25 nmol |  |
| NDR2 | **siRNA1* :** AAGTTACGTCGATCACAACAC | 25 nmol | **F**: CTT GGC TTG GAT GAC TTT GAG  **R** : GCT CTT TTT CAA GCA TAT CAG C |
|  | **siRNA2 :** AAGACACCTTGACAGAAGAGG | 25 nmol |  |
| SDC1 | **siRNA1* :**  CAGGUGCAGGUGCUUUGCAAGAUA U | 25 nmol | **F**: gga gca gga ctt cac ctt tg  **R** : ctc cca gca cct ctt tcc t |
|  | **siRNA2 :**  GCCCACCAAACAGGAGGAAUUCUAU | 25 nmol |  |
|  | ***** siRNA illustrated in this manuscript |  |  |
|  |  |  |  |
|  |  |  |  |
|  |  |  |  |
|  |  |  |  |
|  |  |  |  |

**TableS3**. Antibodies used in this work.

| Antibodies | SOURCE | IDENTIFIER |
| --- | --- | --- |
| Rabbit polyclonal anti-Ect2 (clone C-20) | Santa Cruz Biotechnology | Cat# sc-1005, RRID:AB_2246263 |
| Rabbit polyclonal anti-Fidgetin (clone H-146) | Santa Cruz Biotechnology | Cat# sc-68343, RRID:AB_2104670 |
| Mouse monoclonal anti-Spastin (clone A-2) | Santa Cruz Biotechnology | Cat# sc-271247, RRID:AB_10613446 |
| Mouse monoclonal anti-Katanin p80 B1 (clone C-4) | Santa Cruz Biotechnology | Cat# sc-377226 |
| Mouse monoclonal anti-Citron Kinase (CRIK, clone 6) | Santa Cruz Biotechnology | Cat# sc-136283, RRID:AB_10610237 |
| Mouse monoclonal anti-Anillin (clone B-10) | Santa Cruz Biotechnology | Cat# sc-271814, RRID:AB_10709437 |
| Rabbit polyclonal anti-MKLP-1 (clone N-19) | Santa Cruz Biotechnology | Cat# sc-867, RRID:AB_631959 |
| Mouse monoclonal anti-NDR1 | Santa Cruz Biotechnology | Cat# sc-100404 |
| Rabbit polyclonal anti-Sox9 (clone H-90) | Santa Cruz Biotechnology | Cat# sc-20095, RRID:AB_661282 |
| Mouse monoclonal anti-E-Cadherin (clone 32A8) | Cell Signaling Technology | Cat# 5296S, RRID:AB_10706939 |
| Rabbit monoclonal anti-RhoA (clone 67B9) | Cell Signaling Technology | Cat# 2117, RRID:AB_10693922 |
| Rabbit monoclonal anti-RhoB | Cell Signaling Technology | Cat# 2098S, RRID:AB_2179103 |
| Rabbit monoclonal anti-Rab11a/b (clone D4F5) | Cell Signaling Technology | Cat# 5589S, RRID:AB_10693925 |
| Rabbit monoclonal anti-Rac1/2/3 | Cell Signaling Technology | Cat# 2465S, RRID:AB_10695732 |
| Rabbit monoclonal anti-PRC1 | Cell Signaling Technology | Cat# 3639S, RRID:AB_11178940 |
| Rabbit monoclonal anti-phospho-YAP (Ser127) | Cell Signaling Technology | Cat# 4911S, RRID:AB_2218913 |
| Rabbit monoclonal anti-YAP | Cell Signaling Technology | Cat# 14074S |
| Rabbit polyclonal anti-ZO-1 | Cell Signaling Technology | Cat# 5406S, RRID:AB_1904187 |
| Rabbit monoclonal anti-GEF-H1 (clone 55B6) | Cell Signaling Technology | Cat# 4076S, RRID:AB_10699018 |
| Rabbit monoclonal anti-phospho-GEF-H1 (S885) | Cell Signaling Technology | Cat# 14143S |
| Anti-rabbit IgG, HRP-linked Antibody | Cell Signaling Technology | Cat# 7074, RRID:AB_2099233 |
| Anti-mouse IgG, HRP-linked Antibody | Cell Signaling Technology | Cat# 7072S, RRID:AB_10708988 |
| Mouse monoclonal anti-Tubulin | Sigma-aldrich | Cat# T9028, RRID:AB_261811 |
| Rabbit polyclonal anti-Aurora B | Sigma-aldrich | Cat# A5102, RRID:AB_476740 |
| Mouse monoclonal anti-NDR2 (clone 4D8) | LSBio | Cat# LS-C174201-100 |
| Mouse monoclonal anti-Syntaxin-16 | abcam | Cat# ab134945 |
| Mouse monoclonal anti-RASSF1A | eBioscience | Cat# 14-688-82 |
| Mouse monoclonal anti-N-Cadherin | eBioscience | Cat# 14-3259-82 |
| Mouse monoclonal anti-Cytochrome c | BD Pharma | Cat# 556432, RRID:AB_396416 |
| Goat polyclonal anti-Syndecan-1 (SDC1) | R&D | Cat# AF2780, RRID:AB_442186 |
| Donkey anti-Mouse IgG (H+L) Secondary Antibody, Alexa Fluor 555 | Invitrogen | Fisher Scientific Cat# 10398212 |
| Donkey anti-Rabbit IgG (H+L) Secondary Antibody, Alexa Fluor 488 | Invitrogen | Fisher Scientific Cat# 10424752 |
| Donkey anti-Rabbit IgG (H+L) Secondary Antibody, Alexa Fluor 647 | Invitrogen | Fisher Scientific Cat# 10543623 |
| Donkey anti-Goat IgG (H+L) Secondary Antibody, Alexa Fluor 488 | Invitrogen | Fisher Scientific Cat# 10246392 |
