# Supplementary material for: NDR2 kinase contributes to cell invasion and cytokinesis defects induced by the inactivation of RASSF1A tumor-suppressor gene in lung cancer cells
Source: J Exp Clin Cancer Res. 2019 Apr 12;38:158. doi: 10.1186/s13046-019-1145-8 (PMC6461807; doi:10.1186/s13046-019-1145-8)
Supplement: Supplementary file 4 — Figure S1. NDR1 or NDR2 depletion abolishes mobility and metastasis properties in HBEC with RASSF1A depletion without lead to cells death. Figure S2: NDR2 depletion abolishes mobility and metastasis properties in HBEC with RASSF1A depletion without lead to cells death. Figure S3. NDR1 or NDR2 restores mobility and 3D migration in HBEC with respectively NDR1 or NDR2 depletion. Figure S4. NDR1/2 expression in shcontrol, shNDR1 or shNDR2 HBEC. Figure S5. NDR1orNDR2depletionpartlyabolishedEMTinRASSF1A-depletedH1299cells. Figure S6. NDR2depletionabolishesYAPactivationinRASSF1A-depletedA549orH1650cells. Figure S7. TheheparansulfateproteoglycanSyndecan-1(SDC-1)mediatestheinteractionandphosphorylationofGEF-H1byNDR2inHBEC-3cells. Figure S8. Sanger analysis of GEF-H1 S265A or S885A mutants. Figure S9. RASSF1A is not required for cleavage plan or equatorial structure of HBEC. Figure S10. RASSF1Adepletion induces defects on metaphase and anaphase and is required for mid body formationin HBEC cells. Figure S11. RASSF1A depletion leads to accumulation of enzymes involved in midbody cut (A) and alterations in the content of proteins crucial for intracellular traffic and mitosis (B) in HBEC cells. Figure S12. GEF-H1 silencing mimics cytokinesis failure induced by RASSF1A loss in HBEC-3 cells while NDR2 depletion in RASSF1A-depleted H1299 cells restores proper cytokinesis. Figure S13. RASSF1A/RhoB/GEF-H1/NDR2 mRNA impacts on survival from of 681 patients with NSCLC,TCGAcohort. (PDF 2685 kb) [file 13046_2019_1145_MOESM2_ESM.pdf]

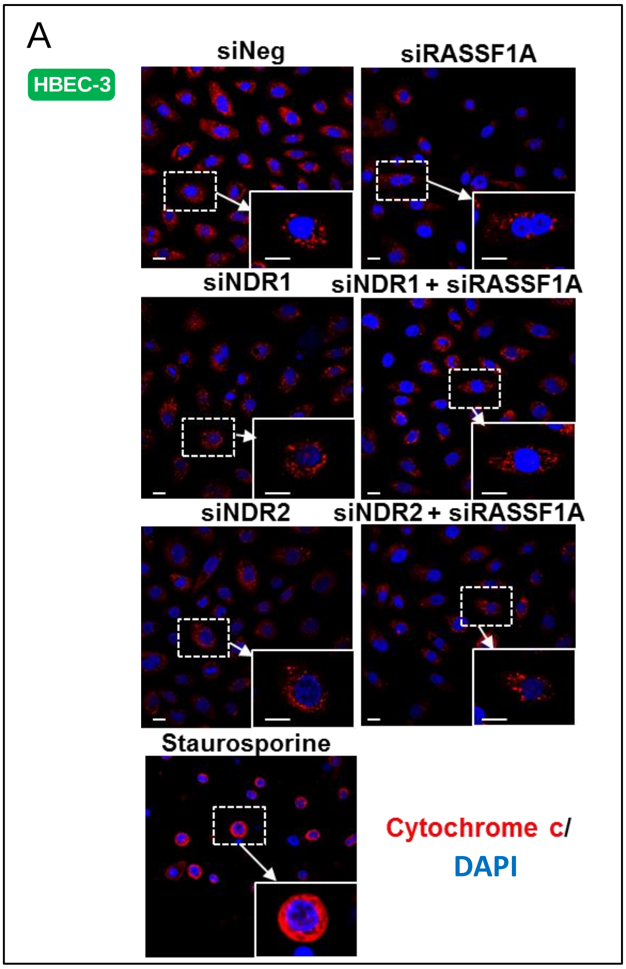

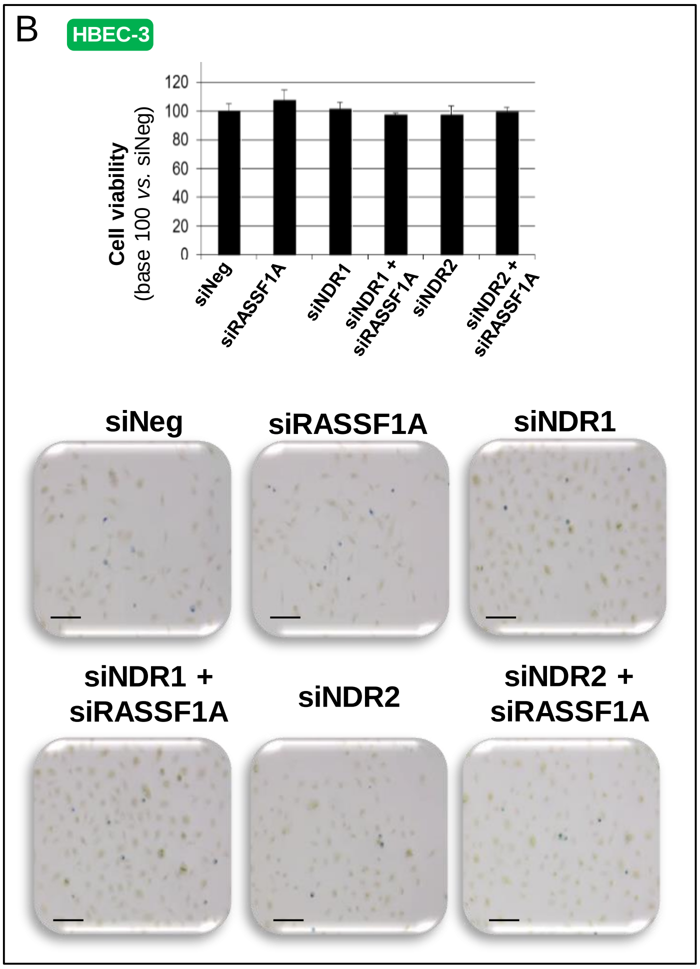

Figure S1. NDR1 or NDR2 depletion abolishes mobility and metastasis properties in HBEC with RASSF1A depletion without lead to cells death.

HBEC-3 were transfected with non-silencing siRNA (siNeg), siRASSF1A and/or with siNDR1 or siNDR2. Experiences were performed 48 hours after transfection.

A-B) HBEC-3 cells death was measured by evaluating Cytochrome c releasing **(A)** and quantifying cell viability with Trypan blue coloration **(B)**. **A-B)** Representative pictures are presented.

For all histograms, error bars indicate the SEM of at least three independent experiments. *P<0.05, **P<0.01 and ***P<0.001, using an ANOVA test followed by Dunnett test.

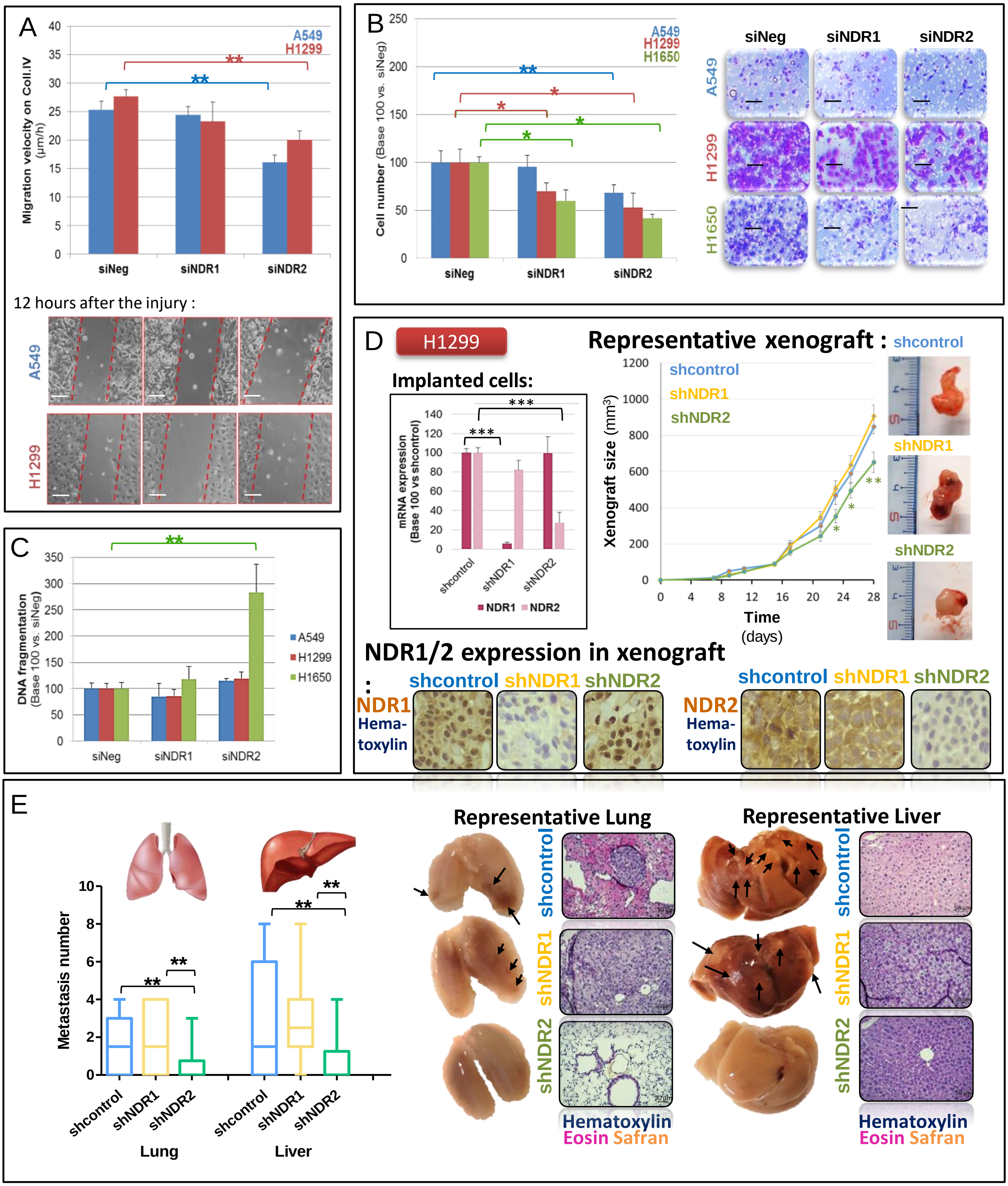
A
Migration velocity on Coll.IV (µm/h)
A549
H1299
siNeg
siNDR1
siNDR2
12 hours after the injury :
B
Cell number (Base 100 vs. siNeg)
H1650
C
DNA fragmentation (Base 100 vs. siNeg)
D
H1299
Implanted cells:
mRNA expression (Base 100 vs shcontrol)
shcontrol
shNDR1
shNDR2
NDR1
NDR2
Representative xenograft : shcontrol
Xenograft size (mm³)
Time (days)
NDR1/2 expression in xenograft :
Hema-toxylin
E
Metastasis number
Lung
Liver
Representative Lung
Representative Liver
Hematoxylin
Eosin Safran

Figure S2

Figure S2: NDR2 depletion abolishes mobility and metastasis properties in HBEC with RASSF1A depletion without lead to cells death. A-C) H1299, A549 and H1650 were transfected with siNeg, siRASSF1A and/or with siNDR1 or siNDR2. Experiments were performed 48 hours after transfection. **A**) Wound healing assay of transfected A549 or H1299 cells on collagen IV coating. Scale bar represents 100 µm. **B**) Invasion capacity of transfected A549, H1299 or H1650 cells on BioCoat Matrigel Invasion Chamber. Relative invasion normalized to that of the cells transfected with siNeg. Scale bar represents 80 µm. **C**) DNA fragmentation of transfected A549, H1299 or H1650 cells. **D-E**) H1299 were transfected with shcontrol, shNDR1 or shNDR2. ShNDR1 or shNDR2-infected H1299 cells suspension (1x10^7 cells in 0.1ml of Matrigel®) were injected subcutaneously in male Fox Chase SCID$^{-/-}$ Beige mice (Ten mice per group). **D**) Xenograft tumor size [length (L)/width (l)/thickness (e)]. Expression levels for NDR1 and NDR2 of the injected cells are presented on the left of the xenografts growth curves. Representative xenograft and representative expression of NDR1 or NDR2 assayed by immunohistochemistry on the shNDR1 or NDR2 xenograft are presented respectively on the right of and under the xenografts growth curves. **E**) Quantification of lung and liver microscopic nodules metastases for H1299 cells expressing suNDR1, shNDR2 or shcontrol. Excised mice lung and liver as histologic photographs of the lung and liver metastases after injection with shNDR1, shNDR2 or shcontrol are presented below the quantification. For all histograms, error bars indicate the SEM of at least three independent experiments. *P<0.05, **P<0.01 and ***P<0.001, using an ANOVA test followed by Dunnett test.

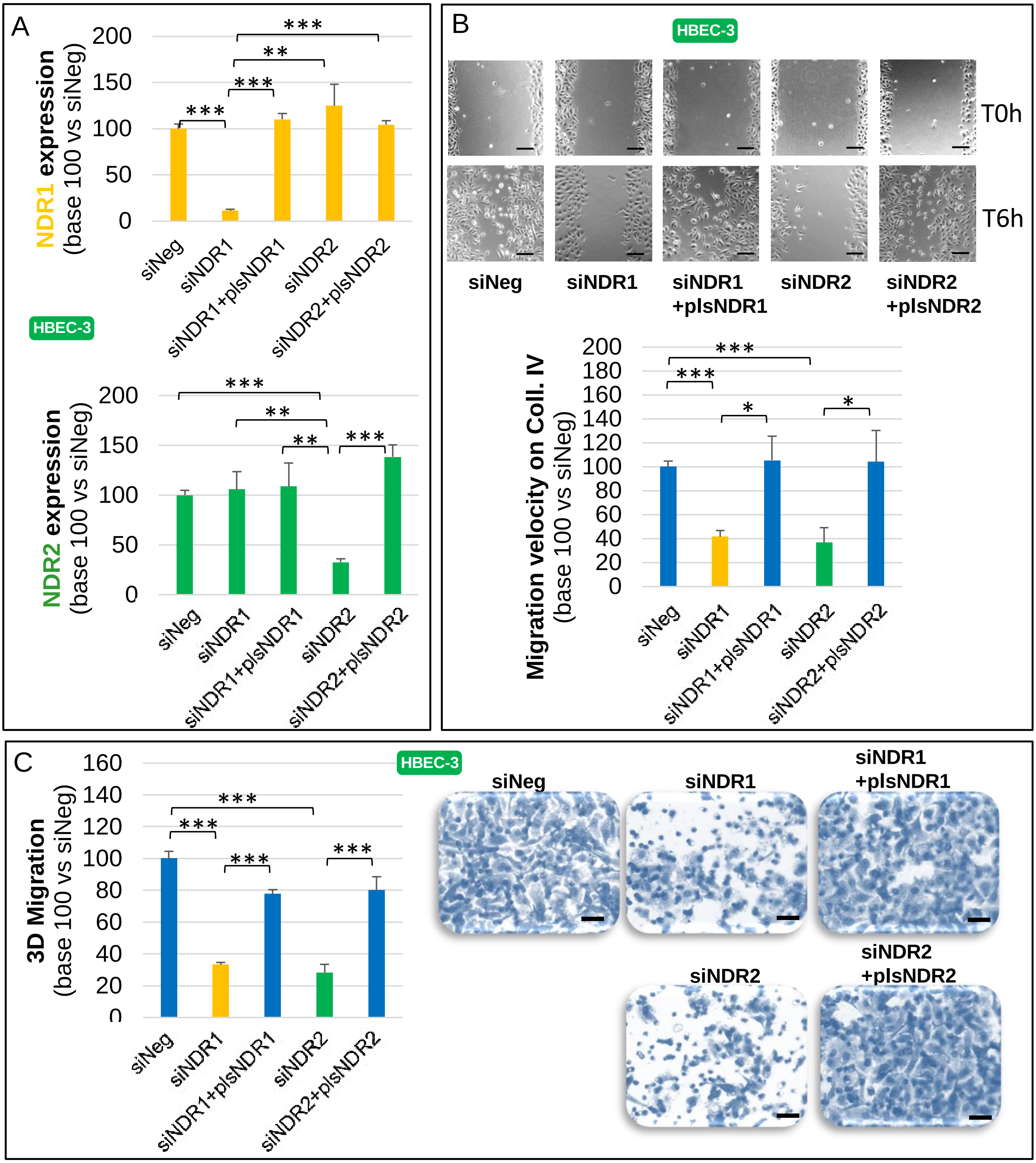

Figure S3. NDR1 or NDR2 restores mobility and 3D migration in HBEC with respectively NDR1 or NDR2 depletion. A-C) HBEC-3 cells were transfected with siNeg, siNDR1, siNDR1+plsNDR1, siNDR2 or siNDR2+plsNDR2. Experiments were performed 48 hours after transfection. **A**) NDR1 or NDR2 mRNA expression according the transfection condition assayed by RT-PCR. **B**) Wound healing assay of transfected HBEC-3 cells on collagen IV coating. Scale bar represents 100 µm. **C**) 3D Migration of transfected HBEC-3 cells on Boyden Chamber. Relative migration normalized to that of the cells transfected with siNeg. Scale bar represents 50 µm. For all histograms, error bars indicate the SEM of at least three independent experiments. *P<0.05, **P<0.01 and ***P<0.001, using an ANOVA test followed by Dunnett test.

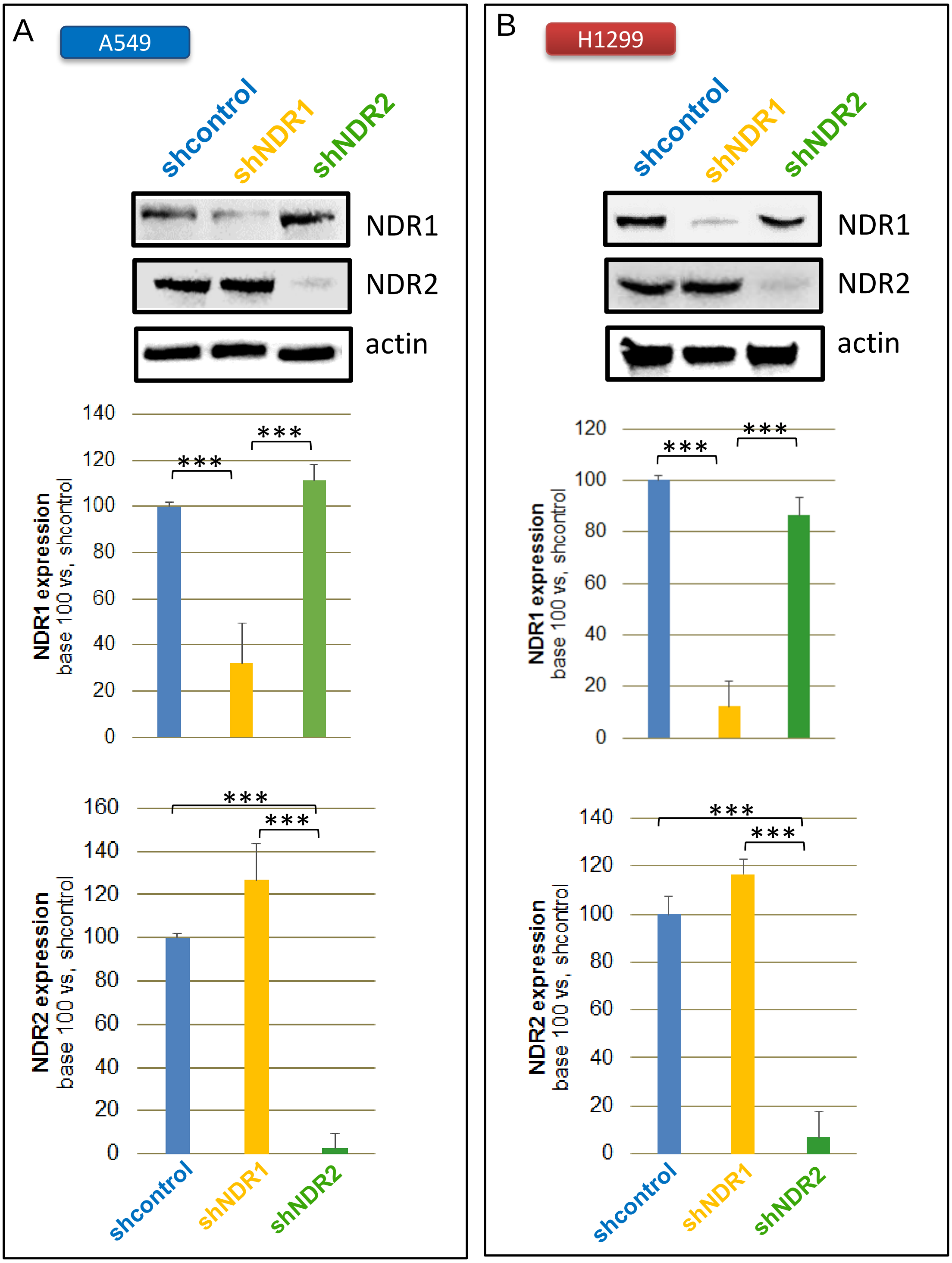

Figure S4. NDR1/2 expression in shcontrol, shNDR1 or shNDR2 HBEC.
A549 (**A**) or H1299 (**B**) cells were transfected with shcontrol, shNDR1 or shNDR2. Quantification of NDR1, NDR2 or actin by western blot were performed on all protein extract.
For all histograms, error bars indicate the SEM of at least three independent experiments. *P<0.05, **P<0.01 and ***P<0.001, using an ANOVA test followed by Dunnett test.

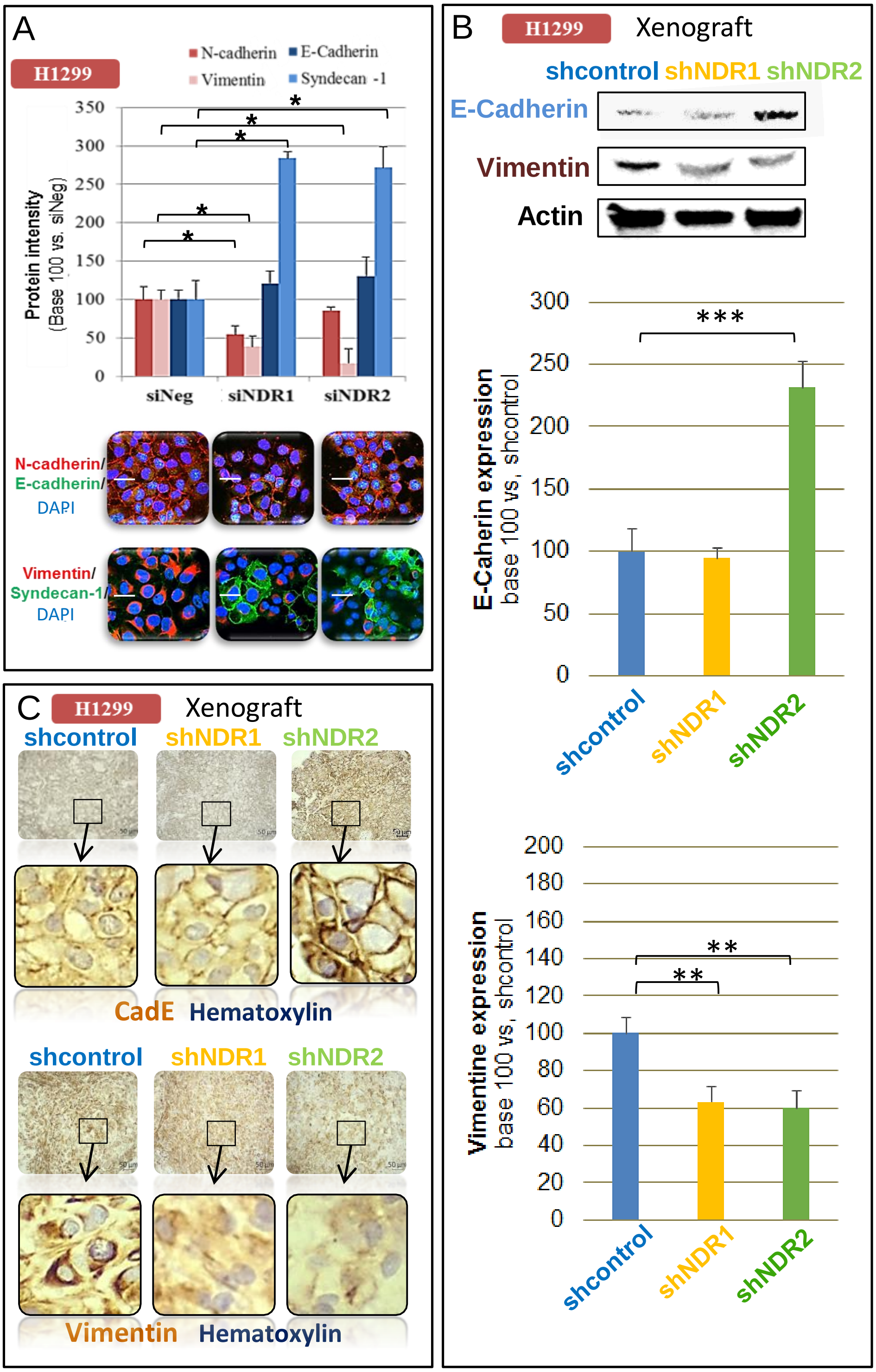

Figure S5. **NDR1 or NDR2 depletion partly abolishes EMT in RASSF1A-depleted H1299 cells.**

A) H1299 cells were transfected with siNeg, siRASSF1A and/or with siNDR1 or siNDR2.

B-C) Xenograft obtained from shcontrol, shNDR1 or NDR2 H1299 cells.

Quantification of E-Cadherin, syndecan-1, ZO-1, vimentin and/or N-cadherin by Immunofluorescence (**A**), western blot (**B**) or immunohistochemistry (**C**).

Error bars indicate the SEM (n≥3). *P<0.05, **P<0.01 and ***P<0.001, using an ANOVA test followed by Dunnett's test.

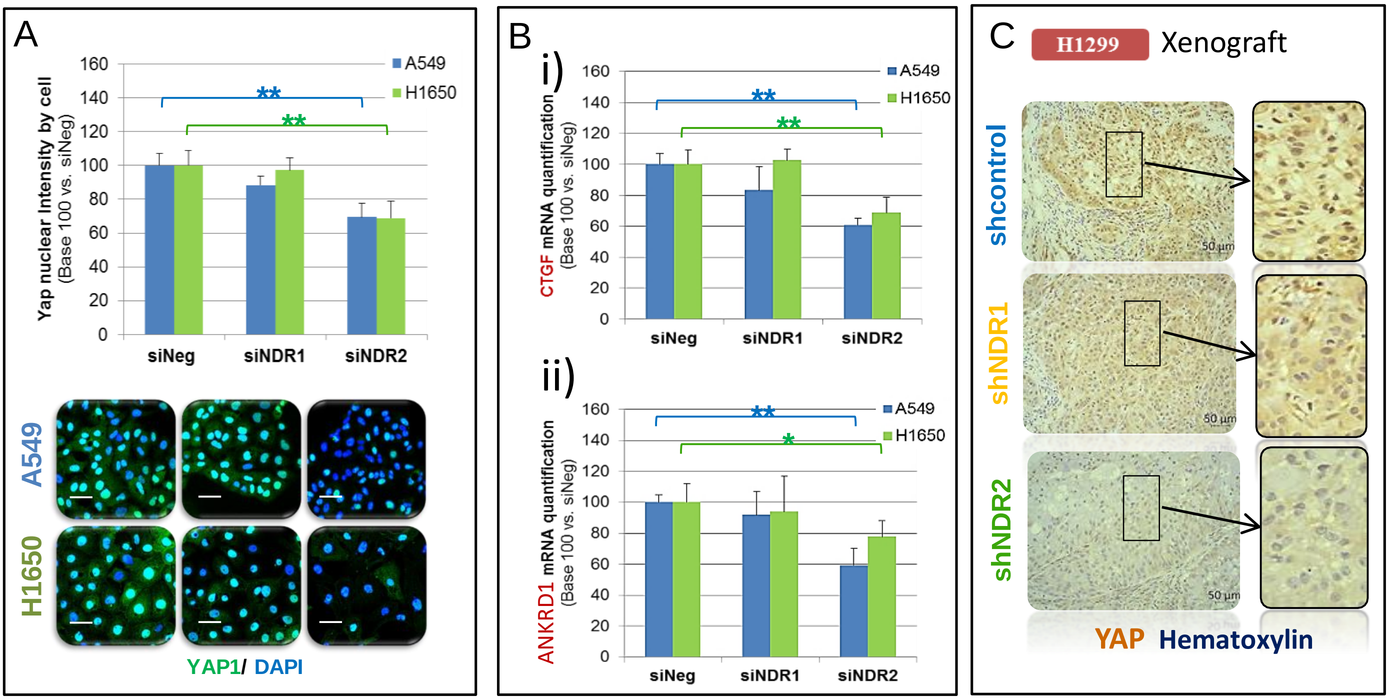

Figure S6. NDR2 depletion abolishes YAP activation in RASSF1A-depleted A549 or H1650 cells.

A-B) H1650 or A549 cells were transfected with siNeg, siRASSF1A and/or with siNDR1 or siNDR2.

C) Xenograft obtained after subcutaneous injection of shcontrol, shNDR1 or NDR2 H1299 cells.

Nuclear YAP quantification by immunofluorescence (**A**) or by immunohistochemistry (**C**)

B) Quantification of CTGF (**Bi**) & ANKRD1 (**Bii**) mRNA using actin as an internal control in H1650 and A549 cells.

Error bars indicate the SEM (n≥3). *P<0.05, **P<0.01 and ***P<0.001, using an ANOVA test followed by Dunnett's test.

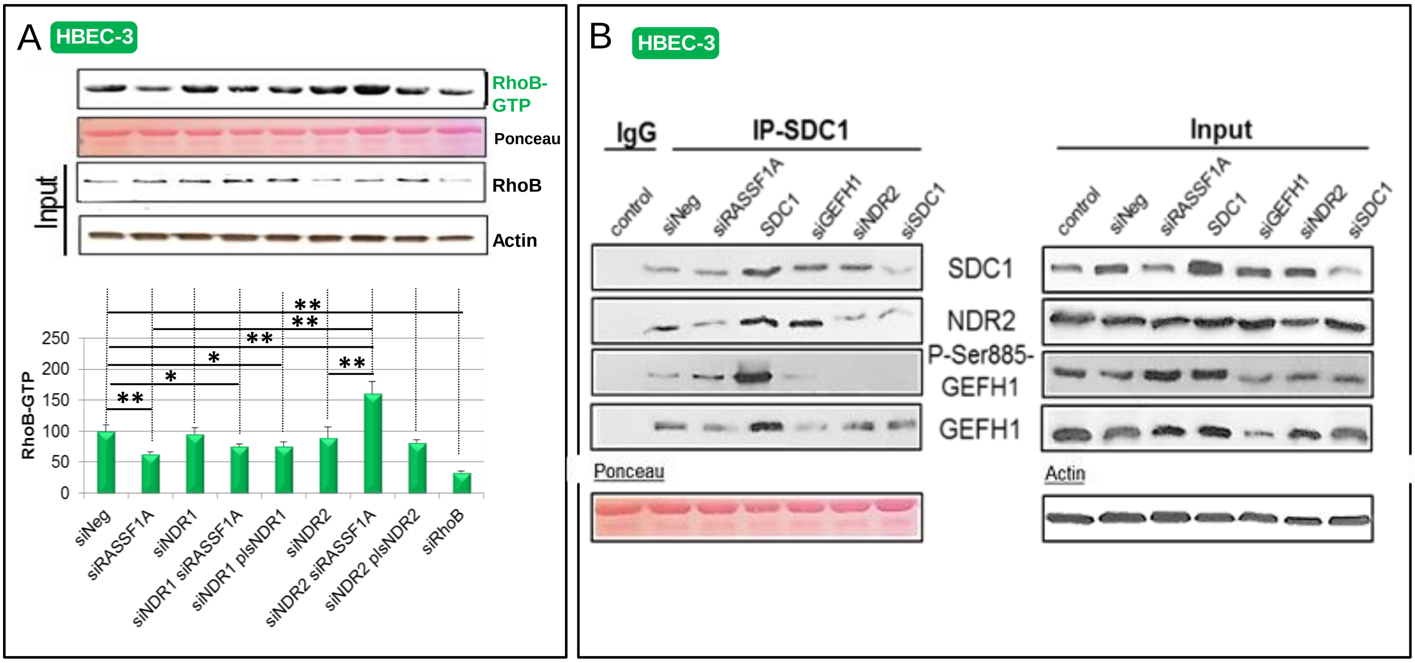

Figure S7. The heparan sulfate proteoglycan Syndecan-1 (SDC-1) mediates the interaction and phosphorylation of GEF-H1 by NDR2 in HBEC-3 cells.

HBEC-3 cells were transfected with siNeg and/or siRASSF1A, siNDR1, siNDR2, siGEF-H1, siSyndecan-1 (SDC1), pcDNA3-NDR1, pcDNA3-NDR2 or pcDNA3-SDC1.

A) GST-RBD pull-down assay in HBEC-3 cells using siRhoB as control.

B) Immunoprecipitation performed on HBEC-3 protein extracts with anti-SDC1. Proteins immunoprecipitated were revealed by western blot, using anti-GEFH1 (P885 or not), anti-SDC1, anti-NDR2 antibodies. Actin was used as internal control for Input and Ponceau staining for Immunoprecipitation.

Error bars indicate the SEM (n≥3). *P<0.05, **P<0.01 and ***P<0.001, using an ANOVA test followed by Dunnett's test

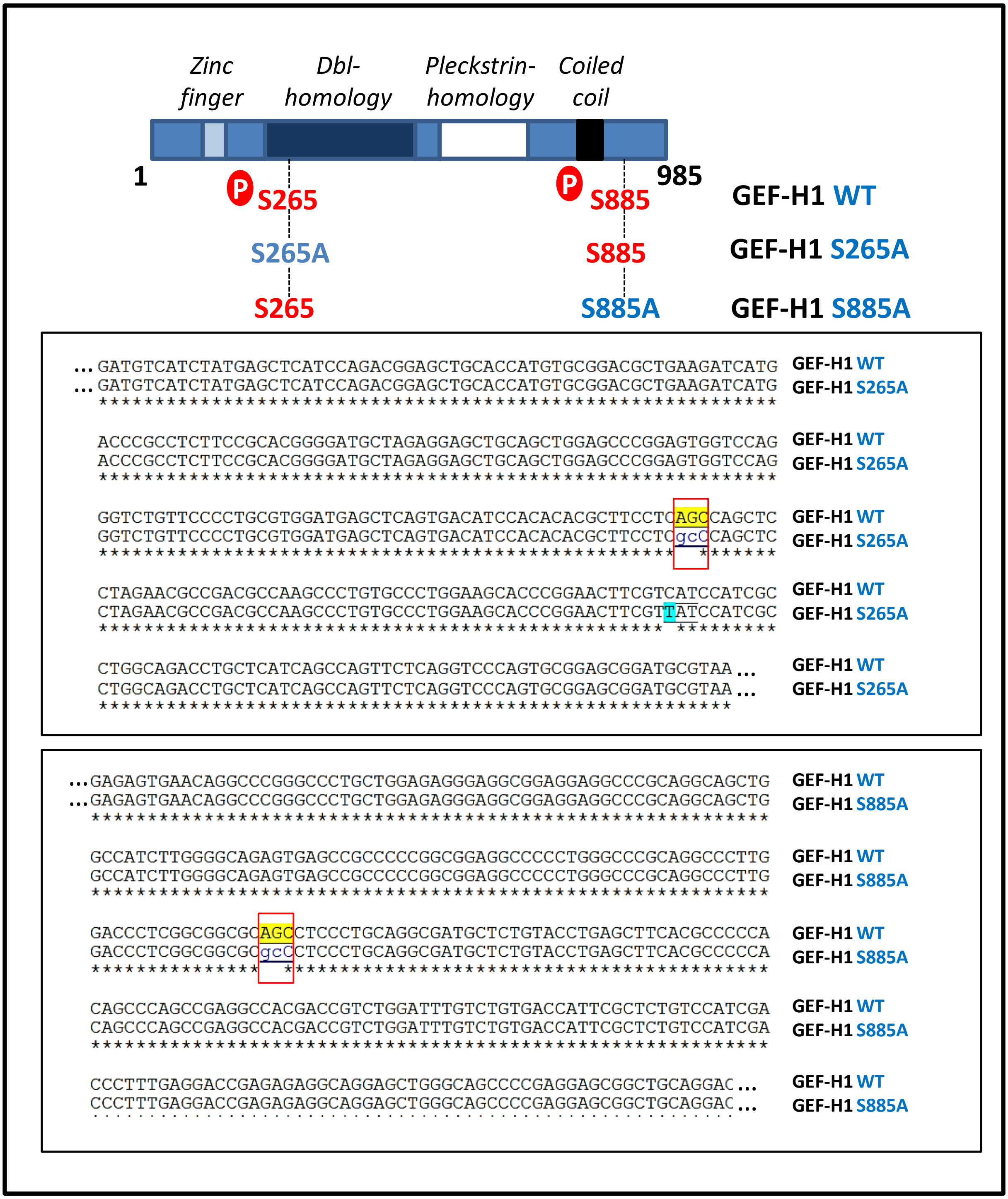

Figure S8. Sanger analysis of GEF-H1 S265A or S885A mutants.
Plasmids with the mutated sequence for GEF-H1 on Ser265 or 885 was confirmed by Sanger analysis

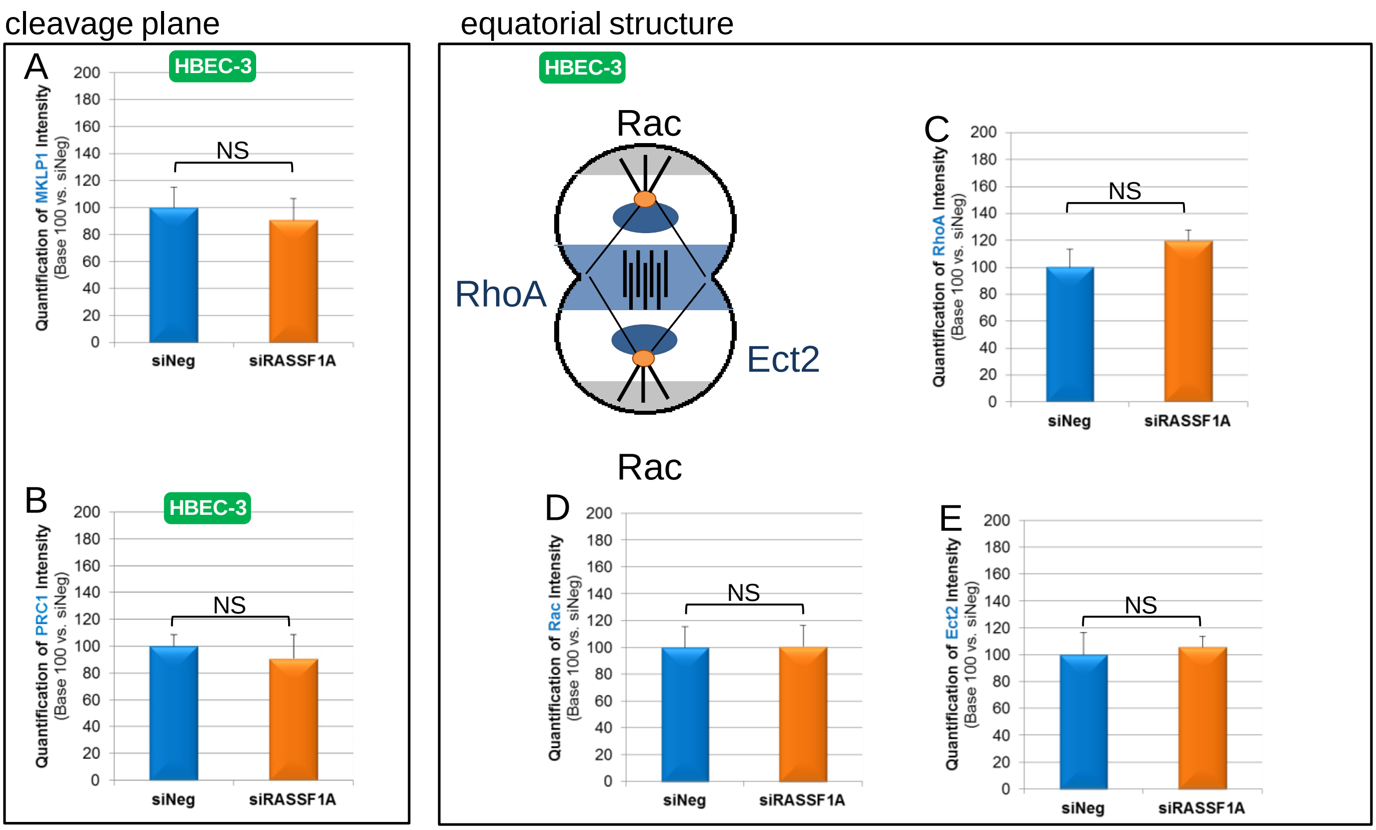

Figure S9. RASSF1A is not required for cleavage plan or equatorial structure of HBEC.
HBEC-3 or HBEC-3-RasV12 cells were incubated with siRASSF1A or siNeg (control). The photographs were obtained 48 hours after transfection. At 48 h post-transfection, cells were fixed and stained with Anti-MLKP1 (**A**) or Anti-PRC1 (**B**) for monitoring cleavage plane, with Anti-RhoA (**C**), Anti-Rac (**D**), or Anti-Ect2 (**E**) for monitoring equatorial structure.

A-E) Nuclei were paint with Dapi. Scale bar represents 50 µm.

For all histograms, error bars indicate the standard error of the mean (SEM) of at least three independent experiments. *P<0.05, **P<0.01 and ***P<0.001, using a t-test.

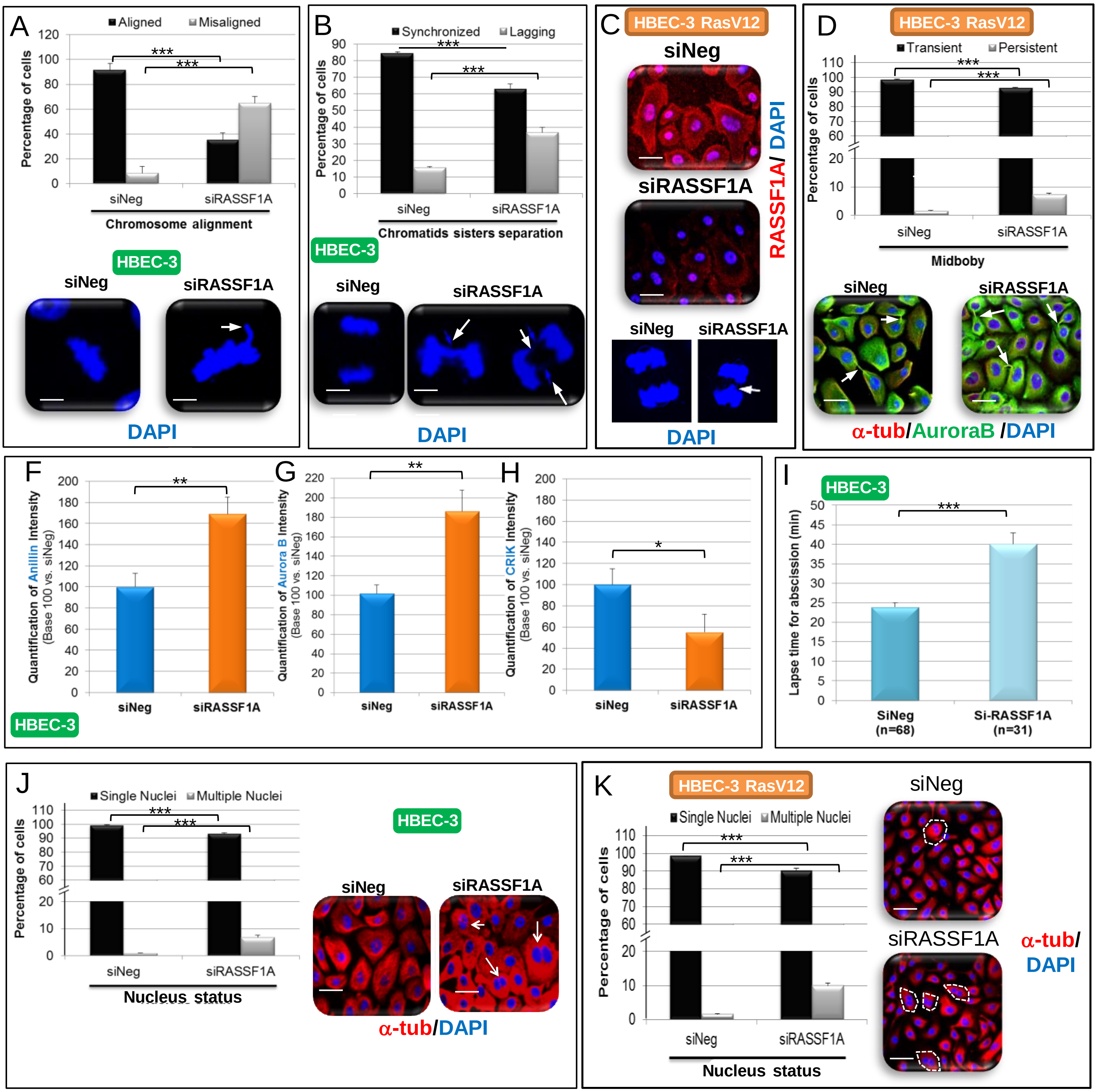

Figure S10. RASSF1A depletion induces defects on metaphase and anaphase and is required for midbody formation in HBEC cells. HBEC-3 or HBEC-3-RasV12 cells were incubated with siRASSF1A or siNeg (control). The photographs were obtained 48 hours after transfection. At 48 h post-transfection, cells were fixed and stained with Anti-RASSF1A (red), Anti-tubulin (red or green in **D**) and DAPI (blue). The number of cells with misaligned chromosome during metaphase (**A:** HBEC-3), chromosome lagging during anaphase (**B:** HBEC-3, **C:** HBEC-3-RasV12), the persistent midbody in HBEC-3 RasV12 cells following Anti-tubulin (red) and Anti-Aurora-B (green) co-staining (**D**), the expression of Anillin (red, **F**), Aurora-A (green, **G**) or Citron Kinase (red, **H**) for monitoring midbody formation and cells with multiple nuclei (**J:** HEBC-3, **K:** HBEC-3-RasV12) were scoring. **A-F, J-K**) Scale bar represents 50 µm. **I**) Lapse time for abscission was quantified by scoring > 100 cells. For all histograms, error bars indicate the standard error of the mean (SEM) of at least three independent experiments. *P<0.05, **P<0.01 and ***P<0.001, using an ANOVA test followed by Dunnett test.

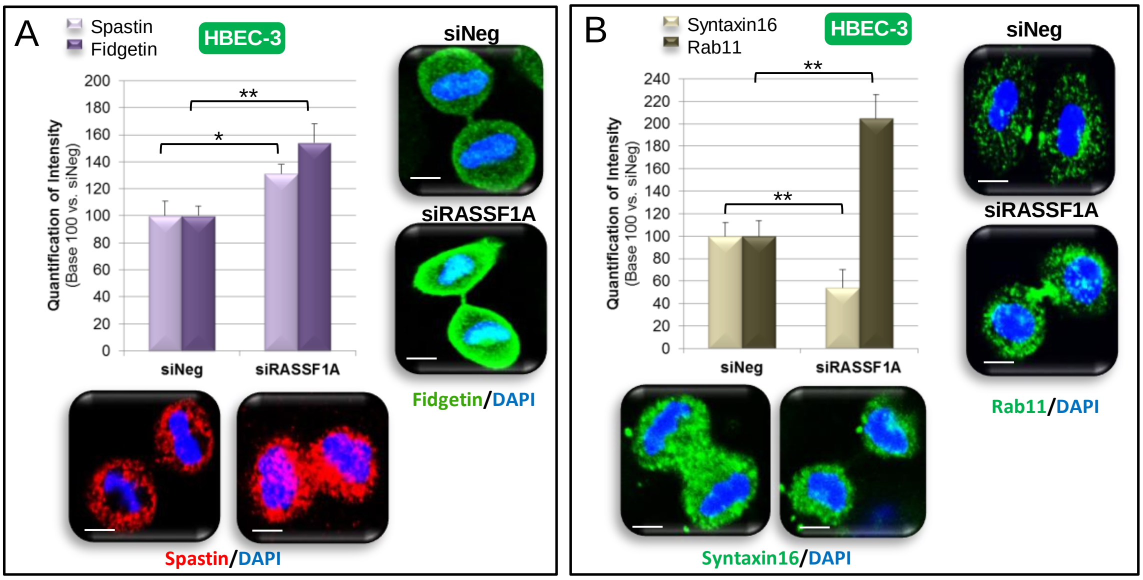

Figure S11. RASSF1A depletion leads to accumulation of enzymes involved in midbody cut (A) and alterations in the content of proteins crucial for intracellular traffic and mitosis (B) in HBEC cells.

HBEC-3 cells were transfected with siRASSF1A or siNeg.

Spastin, Fidgetin (**A**), Rab11 and Syntaxin 16 (**B**) expressions were assayed by immunostaining with DAPI for the nucleus.

A-B) Scale bar represents 50 µm.

Error bars indicate the SEM (n≥3). *P<0.05, **P<0.01 and ***P<0.001, using an ANOVA test followed by Dunnett's test.

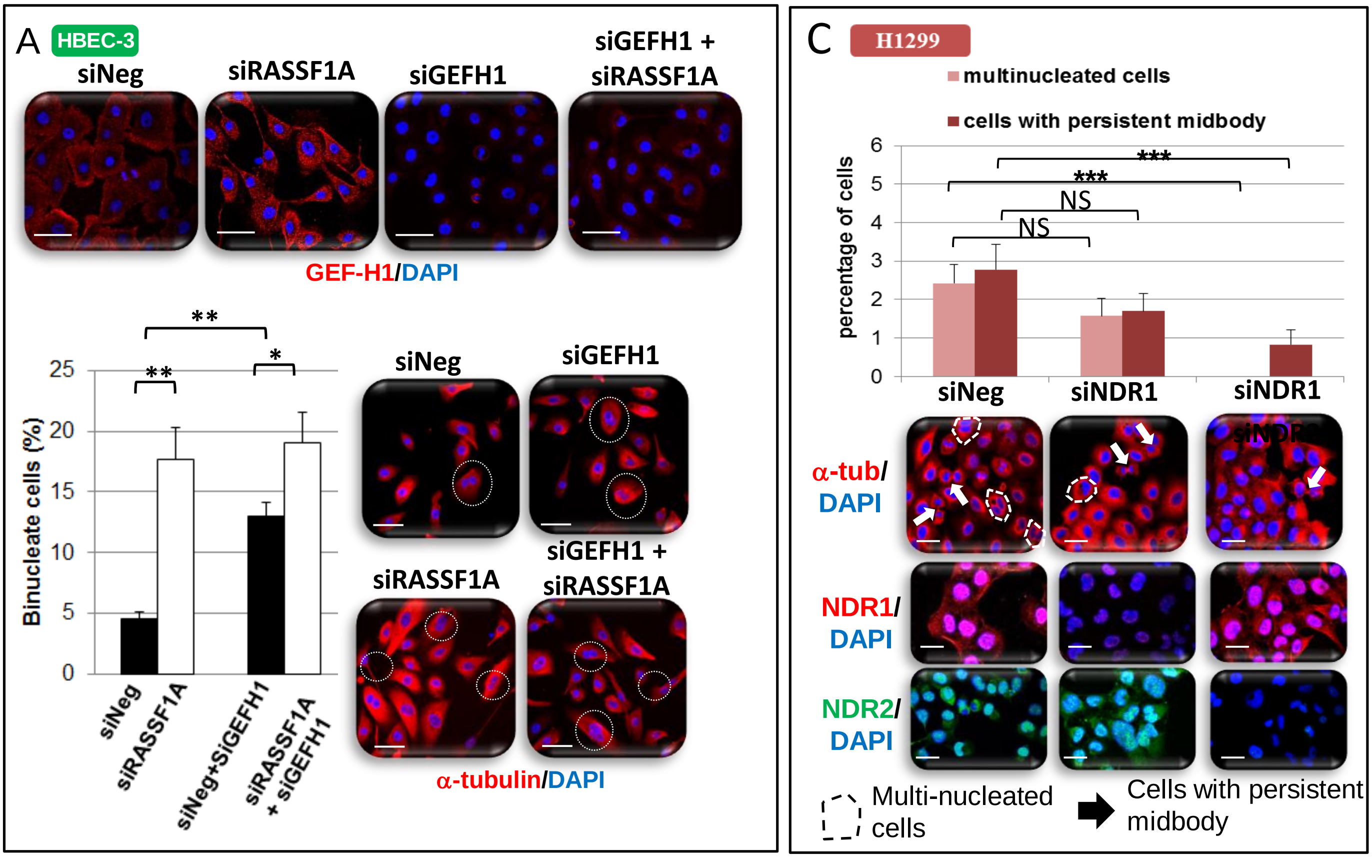

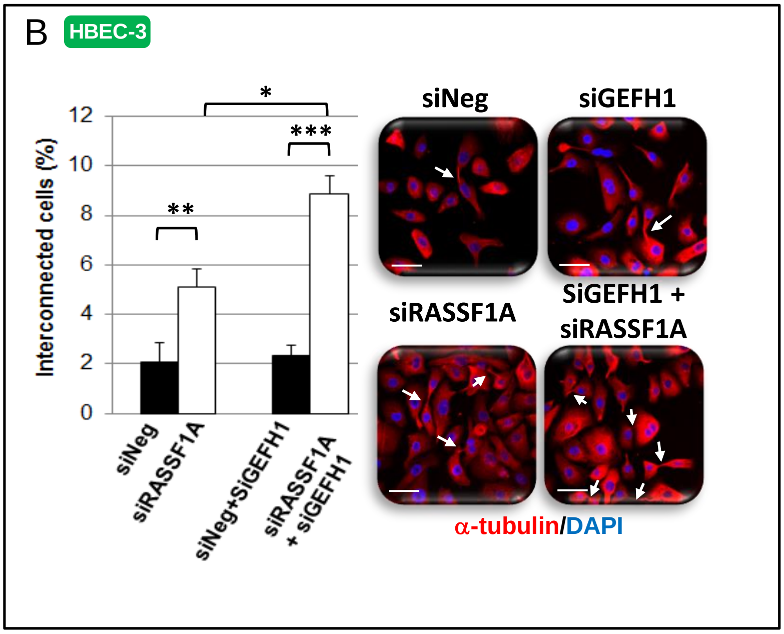

Figure S12. GEF-H1 silencing mimics cytokinesis failure induced by RASSF1A loss in HBEC-3 cells while NDR2 depletion in RASSF1A-depleted H1299 cells restores proper cytokinesis.

HBEC-3 cells (**A-B**) or H1299 cells (**C**) were transiently transfected with non-silencing siRNA (siNeg), siRASSF1A and/or siGEF-H1 (A). The number of binucleate (**A, C**) and interconnected cells (**B, C**) were counted 48 hours after transfection after alpha-tubulin (red) and DNA (blue, DAPI) staining from cells expressing or not GEF-H1 (**A-B**) or NDR1 or NDR2 (**C**). These numbers are expressed as a percentage in control and siRNA-transfected cells. For all histograms, error bars indicate the standard error of the mean (SEM) of at least three independent experiments. *P<0.05, **P<0.01 and ***P<0.001, using an ANOVA test followed by Dunnett's test.

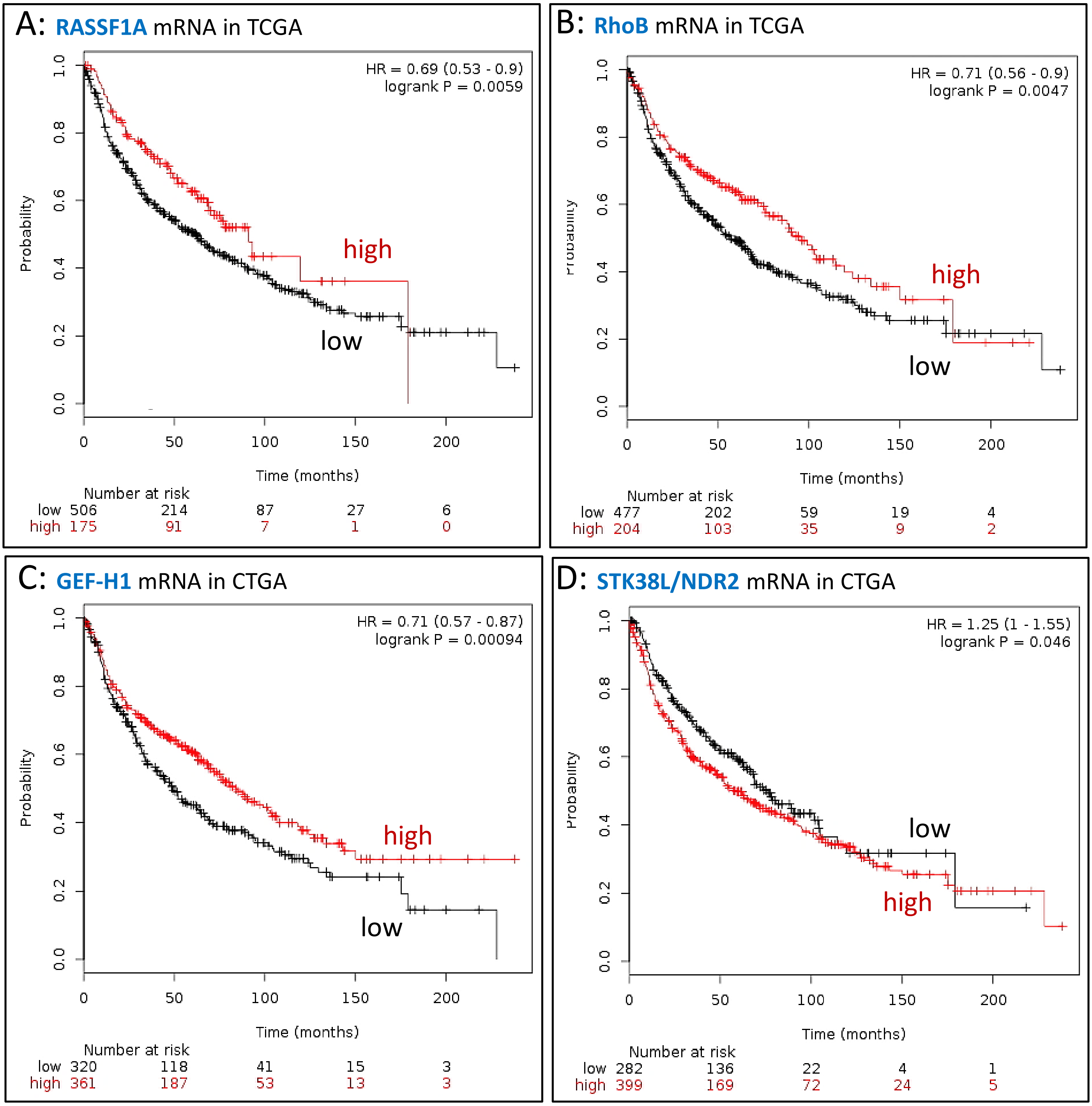

Figure S13. RASSF1A/RhoB/GEF-H1/NDR2 mRNA impacts on survival from of 681 patients with NSCLC, TCGA cohort.
